# Assessment of Genetic Diversity and Population Structure in Oil-Bearing Rose Genotypes Using Start Codon-Targeted (SCoT) Markers

**DOI:** 10.3390/plants15050761

**Published:** 2026-03-01

**Authors:** Mariya Zhelyazkova, Veselina Badzhelova, Florentina Barbu, Stela Lazarova, Peter Hristov

**Affiliations:** 1 Department of Fundamental Sciences in Animal Husbandry, Faculty of Agriculture, Trakia University, Studentski Grad, 6000 Stara Zagora, Bulgaria; 2 Institute for Roses and Aromatic Plants, Agricultural Academy, Boulevard “Osvobojdeni” 49, 6100 Kazanlak, Bulgaria; veselina.nenova@abv.bg; 3 Faculty of Horticulture, University of Agronomic Sciences and Veterinary Medicine of Bucharest, 011464 Bucharest, Romania; flori_barbu@yahoo.com; 4 Institute of Biodiversity and Ecosystem Research, Bulgarian Academy of Sciences, 2 Y. Gagarin Str., 1113 Sofia, Bulgaria; stela.lazarova@gmail.com (S.L.); peter_hristoff@abv.bg (P.H.)

**Keywords:** genetic diversity, Damask rose, essential oil crops, *Rosa damascena*, genetic resources

## Abstract

The oil-bearing rose (*Rosa damascena* Mill.), traditionally cultivated in Bulgaria for centuries, and the rose oil produced from it are of major cultural and economic importance. Its distinctive fragrance and rich aromatic profile are highly valued worldwide. In this study, a set of 15 start codon-targeted (SCoT) molecular markers was used to evaluate the genetic diversity and relationships of 38 rose accessions. The analyzed materials included Bulgarian-bred *R. damascena* cultivars, a locally improved population (‘Population 5’), three oil-bearing species (*Rosa alba* L., *Rosa gallica* L., and *Rosa centifolia* L.), Romanian heritage roses, and an unidentified rose genotype from an old Bulgarian plantation (*Rosa* sp.). The SCoT primers yielded a cumulative count of 238 bands, with an average of 12.9 bands per primer. The range of diversity markers, such as PIC (0.20–0.78), number of different alleles (1.5–2.00), Shannon’s information index (0.24–0.69), and gene diversity (0.15–0.50), provided evidence of genetic differences among the examined accessions. Analysis of Molecular Variance (AMOVA) revealed higher genetic variation within groups (61%) than among the groups (39%). Multivariate analyses (UPGMA, PCoA, and STRUCTURE) resolved the accessions into major genetic clusters corresponding to their taxonomic identity or breeding history. The unidentified *Rosa* sp. formed a distinct genetic group, showing closer affinity to *R. gallica*. The locally improved *R. damascena* ‘Population 5’ exhibited higher genetic diversity than the Bulgarian cultivars. Overall, our results demonstrate the effectiveness of SCoT markers and the value of local and traditional rose germplasm as reservoirs of genetic diversity. The study provides a molecular framework to support breeding, conservation, and sustainable management of oil-bearing rose genetic resources.

## 1. Introduction

Studies on genetic diversity and population structure of *Rosa* species, including cultivated oil-bearing roses, are essential for selection, conservation planning, and the establishment of representative core collections [1,2,3,4]. The identification and preservation of unique genotypes with valuable agronomic or aromatic traits are increasingly important due to recent climate changes, increasing pathogen pressure, and the growing global demand for natural aromatic products [3,5]. Studies aimed at genetic characterization also contribute to clarifying taxonomic uncertainties within the genus, which is well known for its complex evolutionary history, hybridization events, and widespread polyploidy [1,2,3,4,5,6,7]. Moreover, the molecular identification of unknown rose genotypes is becoming particularly important due to the discovery of new or previously neglected populations, especially in regions with rich wild rose diversity [3,4,5,8,9,10,11].

Among the oil-bearing roses, *Rosa damascena* Mill. is the most widely cultivated hybrid worldwide, followed by *Rosa gallica* L., *Rosa centifolia* L., and *Rosa alba* L [12]. *Rosa damascena* is also the most economically significant aromatic and oil-bearing rose, valued for its essential oil, a key ingredient in high-end perfumery, cosmetics, and pharmaceutical products. It has a long cultivation history and significant cultural and symbolic value, reflecting the long-standing relationship between humans and the natural environment [13]. Traditionally cultivated in regions such as Bulgaria, Turkey, Iran, and Morocco, *R. damascena* represents a unique genetic resource shaped by centuries of vegetative propagation, cultural practices, and local environmental conditions [14]. Despite its long history of cultivation, the genetic base of *R. damascena* has often been described as narrow, largely due to predominantly clonal propagation in industrial oil rose plantations [15]. However, advances in molecular genetics over the past two decades have challenged this assumption by revealing previously unrecognized levels of diversity within and among regional populations [8,9,10,11,16,17,18,19].

Native to Europe and parts of western Asia, *R. gallica* was among the first rose species domesticated in Europe and is considered a progenitor of many modern cultivated roses [20]. Also known as French or apothecary’s rose, it has been typically grown for both essential oil production and traditional herbal medicine. Its cultivation is more limited today, usually occurring in small-scale plantations, experimental and research collections, and botanical and historical gardens across parts of Europe, including France, Bulgaria, and other temperate regions [21]. The industrial cultivation of *R. centifolia* is more limited compared with *R. damascena*. It is a complex hybrid; however, its precise parental lineage is not fully resolved. Molecular phylogenetic analyses across the genus *Rosa* have shown that cultivated roses, including old garden types like *R. centifolia*, derive from a mosaic of wild species and multiple hybridization events. Gudin [22] suggests contributions from species such as (*R. gallica* × *Rosa moschata* Herrm.) × *R. alba*, whereas Vukosavljev [23] indicates that it is derived from *R. gallica* and *R. alba* or Damask roses. Both *R. gallica* and *R. centifolia* have also been the subject of recent scientific studies related to their essential oil and hydrosol compositions, underpinning their value as aromatic raw materials and supporting their continued cultivation for niche products and regional aromatic industries [24,25]. *Rosa alba* is also a hybrid of *Rosa canina* L. × *R. gallica* [22] and is mainly grown for aromatic water and local products in parts of North Africa and Europe, including Bulgaria [26].

Of all the roses discussed above, *R. damascena* has also been the most intensively studied species, particularly with regard to its genetic diversity. Early studies used Amplified Fragment Length Polymorphism (AFLP), Inter-Simple Sequence Repeat (ISSR), and microsatellite (SSR) markers, which proved effective for distinguishing between closely related genotypes and assessing population structure [8,16,17,19]. ISSR and start codon-targeted (SCoT) markers have been applied to reveal polymorphism even among accessions previously assumed to be genetically uniform [2,18,19]. More recent advances in SNP-based approaches, including high-throughput sequencing, genotyping-by-sequencing (GBS), and genome-wide analyses, have significantly improved the resolution at which genetic variation can be assessed. These methods enable the detection of fine-scale genetic differences, hidden population structure, as well as applications in quantitative trait loci (QTL) mapping and marker-assisted selection [1,14,27,28]. Integration of molecular and chemical analyses has further demonstrated that genetic variability is often associated with distinct chemotypes and essential oil compositions, suggesting potential for targeted selection and regional product differentiation [18,29].

Another major challenge in rose research is the reliable identification of unknown or mislabelled genotypes. Previous studies have reported the presence of unclassified or previously undescribed genotypes of *R. damascena* with distinct genetic profiles [3,4,8]. These findings highlight the importance of comprehensive and systematic genetic characterization, particularly in regions where traditional cultivation practices or introgression between wild and cultivated populations may have generated genetically divergent lineages [3]. Accurate genotypic classification is also crucial for germplasm conservation, selection programs, and the establishment of authentic geographic indications—an increasingly important issue for countries that rely on rose oil production as a strategic agricultural sector [14,30]. Overall, growing molecular evidence indicates that the genetic diversity of oil-bearing roses is greater and more structured than previously assumed. A comprehensive assessment of this diversity, particularly through the genetic identification of unknown accessions, is therefore essential to enhance conservation efforts, improve selection programs, and fully explore the aromatic and agronomic potential of *R. damascena*.

Among essential oil crops, the rose occupies a leading position and represents one of the most important industrial crops in Bulgaria. Rose oil and rose-derived products are among the most recognizable and emblematic Bulgarian commodities on global markets. Bulgaria has more than 350 years of tradition in the cultivation of the oil-bearing rose, with the earliest documented evidence of rose gardens dating back to 1712 [31]. *Rosa damascena*, also known as the Kazanlik oil-bearing rose in Bulgaria, has been the focus of research at the Institute of Roses, Essential and Medical Cultures (IREMC), Kazanlak, for more than 100 years. As a result of many years of selection efforts, a valuable genetic fund has been created, part of which has been preserved to this day, forming the basis of modern Bulgarian rose production.

In this study, we used SCoT molecular markers to (i) evaluate the genetic diversity of 38 *Rosa* accessions, (ii) clarify the genetic relationships among established *R. damascena* lineages and related species, and (iii) determine the genetic position of an unclassified Bulgarian rose genotype and three old rose plants from Romania.

SCoT markers have been directly linked to gene function and were effectively employed for genotyping and polymorphism assessment [32]; they have been successfully applied in genetic diversity analyses and diagnostic fingerprinting across a wide range of essential oil crops [2,18,33,34,35,36,37].

## 2. Materials and Methods

### 2.1. Plant Material

The plant material was collected in May 2025 and included 35 samples of oil-bearing rose species and cultivars from Bulgaria and three rose accessions from Romania. All Bulgarian plant samples are from the experimental collection of the Institute of Roses, Essential and Medical Cultures (IREMC), Kazanlak. During a field survey conducted in 2023, an old private plantation (over 50 years old) with an unknown oil-bearing rose (*Rosa* sp.) was discovered near the town of Klisura, Bulgaria. Eight specimens were collected and transferred to the IREMC and clonally propagated for further investigations. Plant material from each specimen was included in the present study (designated as Kll–Kl8). Nine samples (P1–P9) from an improved selection of local populations of *Rosa × damascena* nothof. × trigintipetala (Dieck) R.Keller (‘Population 5’); four Bulgarian cultivars—‘Iskra’ (I1–I2), ‘Yanina’ (Y1–Y2), ‘Eleina’ (E1–E2), and ‘Svezhen’ (SV1–SV2); three clones from local populations of *R.× alba* (A1–A3); two specimens (R1–R2) of the Russian cultivar ‘Raduga’, a complex hybrid between a variety of *R. gallica* and *R. damascena*; three accessions of *R. gallica* (G1–G3), and two *R. centifolia* L. (C1–C2) plants were included in the study.

The Romanian samples comprise two accessions (G4 and D) provisionally identified as *R. gallica*—one from Comuna Poeni, Teleorman (44°24′ N, 25°20′ E), and the other from Carbunești, Prahova (45°13′14″ N, 26°12′30″ E)—and one accession from *R. alba* (A4) collected in Carbunești, Prahova (45°13′14″ N, 26°12′30″ E), and introduced in the Faculty of Horticulture, University of Agronomic Sciences and Veterinary Medicine of Bucharest, for further investigation and provided to us for this study. All of these samples originate from old rose plants cultivated in private gardens and used for food products (e.g., jam and herbal tea preparation).

**Figure 1:**
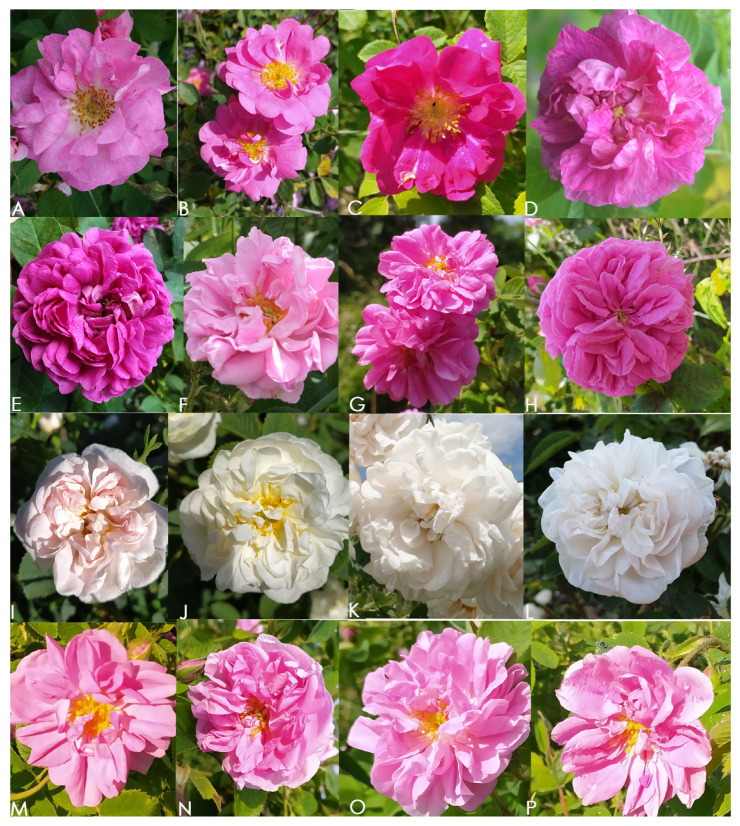
Rose accessions used in the study: (**A**) *Rosa* sp. (Kll–Kl8), (**B)** *Rosa gallica* (G1), (**C)** *R. gallica* (G2–G3), (**D)** *R. gallica* (G4), (**E**) *R. gallica* (D), (**F**) *Rosa damascena* ‘Population 5’ (P1–P9), (**G**) *Rosa* ‘Raduga’ (R1–R2), (**H**) *Rosa centifolia* (C1–C2), (**I**) *Rosa alba* (A1), (**J**) *R. alba* (A2), (**K**) *R. alba* (A3), (**L**) *R. alba* (A4); *R. damascena* cultivars: (**M**) ‘Iskra’ (I1–I2), (**N**) ‘Yanina’ (Y1–Y2), (**O**) ‘Eleina’ (E1–E2), (**P**) ‘Svezhen’ (SV1–SV2).

Detailed characteristics of the sampled plants are presented in Table 1. Representative specimens differing in morphology, species, or cultivar are shown in Figure 1.

Descriptions of *Rosa* accessions: A1–A3 and G1–G3 were provided by V.B. (co-author); G4, D, and A4 by F.B. (co-author); P1–P9 [38] and R1–R2, C1–C2, I1–I2, Y1–Y2, E1–E2, and SV1–SV2 [39] were taken from previously published sources.

### 2.2. Genomic DNA Isolation

Frozen plant tissue from young leaves was homogenized using ZR BashingBead™ Lysis Tubes (Zymo Research Corp., Irvine, CA, USA) with Lysis Buffer in combination with a Disruptor Genie homogenizer (Scientific Industries, Inc., Bohemia, NY, USA). Genomic DNA was subsequently extracted following the manufacturer’s protocol for the GeneMATRIX Plant & Fungi DNA Purification Kit (EURx Ltd., Gdansk, Poland). All DNA samples were adjusted to a working concentration of 20 ng/µL. DNA purity was assessed using a NanoVue Plus spectrophotometer (GE Healthcare UK Limited, Amersham Place, Little Chalfont, Buckinghamshire, UK), and only samples with an A260/280 ratio between 1.8 and 2.0 were used for downstream analyses. The extracted DNA was stored at −20 °C prior to analysis.

### 2.3. PCR Amplification and Visualization

A total of 30 SCoT primers designed by Invitrogen (Invitrogen, Darmstadt, Germany) and selected from previous studies of roses [2,40] were initially tested. Fifteen primers that produced clear, reproducible, and polymorphic banding patterns were ultimately used for amplification across all *Rosa* accessions (Table 2). PCR amplifications were performed in a final volume of 20 µL containing 1 µL genomic DNA (20 ng), 10 µL Red Taq DNA Polymerase 2× Master Mix (1.5 mM MgCl_2_), 1 µL primer (10 pmol), and 8 µL ddH_2_O. All PCR reactions were carried out using a Doppio Gradient 2 × 48-well thermal cycler (VWR^®^, Darmstadt, Germany) under the following conditions: initial denaturation at 94 °C for 5 min; 35 cycles of denaturation at 94 °C for 45 s, primer annealing at 50–58 °C for 45 s, and extension at 72 °C for 90 s; followed by a final extension at 72 °C for 10 min.

PCR products were visualized on 1.7% agarose gels prepared in 1× TBE buffer and stained with GelRed^®^ (Biotium, Fremont, CA, USA) and visualized under UV light using a UV transilluminator (Bio-Imaging System, Modi’in-Maccabim-Re’ut, Israel). Fragment sizes of SCoT-PCR products were estimated using the NZYDNA Ladder VI (NZYtech Lda., Lisbon, Portugal), ranging from 50 to 1500 bp.

### 2.4. Data Analysis

SCoT-amplified fragments were scored as a binary data matrix, recording the presence (1) or absence (0) of each band. The discriminatory capacity of the primers was evaluated through the calculated values of Polymorphic Information Content (PIC) using the formula PICi = 2fi(1 − fi), where i is the locus, fi is the frequency of the amplified fragments, and (1 − fi) is the frequency of the non-amplified fragments [41]; effective multiplex ratio (EMR), calculated using the formula EMR = n × β, where n = mean number of fragments amplified per primer and β = PB/(PB + MB), where PB represents polymorphic fragments and MB represents monomorphic fragments [42]; marker index (MI) calculated using the formula MI = EMR × PIC [43]; and resolving power (RP) calculated using the formula RP = Σ Ib, where Ib = 1 − (2 |0.5 − pi|) and pi were genotypes showing the presence of the fragment [44].

Genetic diversity per locus/primer, represented by Nei’s gene diversity [45] and Shannon’s information index (I), was estimated using PopGen 1.32.

GenAlEx 6.5 [46] was employed to assess population-level genetic diversity, Principal Coordinate Analysis (PCoA), and an Analysis of Molecular Variance (AMOVA). Genetic differentiation among populations was estimated using *Φ*_PT_ (PhiPT) (an *F*_ST_ analogue for dominant markers) via AMOVA. The first three principal coordinate axes (PC1 vs. PC2, PC2 vs. PC3) of Genalex were used for visualization in GraphPad Prism 10.6.1. Structure (v 2.3.4) software was used to analyze population structure using a Bayesian mathematical model for calculating individual genetic similarity weight values (*Q* value) and assessing gene flow [47,48]. The number of clusters (K) was explored across a range from 2 to 11, with 20 independent runs per K value, using a burn-in of 100,000 iterations followed by 10,000 Markov Chain Monte Carlo (MCMC) iterations after burn-in. The optimal K value was determined according to the highest ΔK likelihood, calculated in StructureSelector https://lmme.ac.cn/StructureSelector/ (accessed on 7 October 2025) [49].

A dendrogram based on the Unweighted Pair Group Method with Arithmetic Mean (UPGMA) was constructed in MEGA 12 [50] using the Nei’s genetic distance matrix generated in PopGen 1.32. The resulting cluster tree was graphically refined using iTOL [51].

## 3. Results

### 3.1. SCoT-PCR Amplification Results

**Figure 2:**
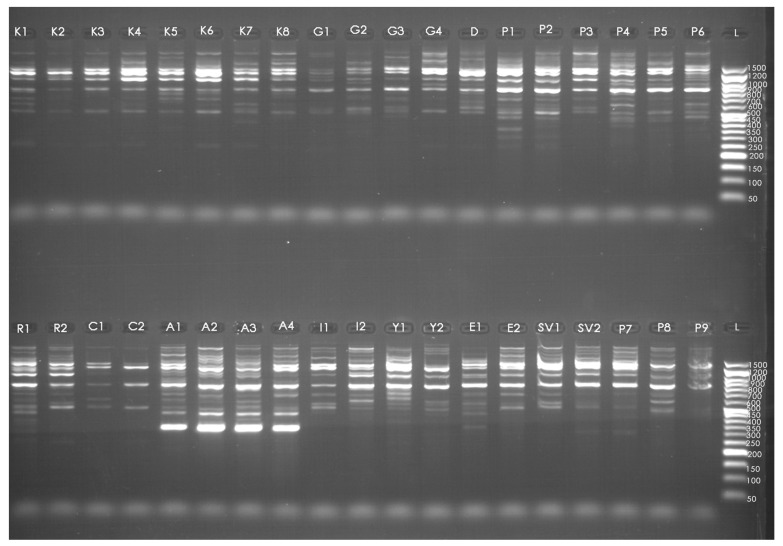
Molecular profiles of 38 *Rosa* accessions in SCoT 25 primer. Lanes marked as 1 to 38 represent the accessions according to serial numbers Kll–Kl8—*Rosa* sp., G1–G4, D—*Rosa gallica*, P1–P9—*Rosa damascena* ‘Population 5’, R1–R2—*Rosa* ‘Raduga’, C1–C2—*Rosa centifolia*, A1–A4—*Rosa alba*, and *R. damascena* cultivars: I1–I2—‘Iskra’, Y1–Y2—‘Yanina’, E1–E2—‘Eleina’, SV1–SV2 –‘Svezhen’, L—Ladder 50–1500 bp.

Fifteen SCoT markers amplified a total of 238 bands, of which 194 were polymorphic and 44 were monomorphic, resulting in an average polymorphism of 89%. Two primers—SCoT 6 and SCoT 21—exhibited a 100% level of polymorphism (Table 3). Across all primers, the number of amplified bands ranged from 2 to 19, with an average of 12.9 bands. The maximum number of polymorphic bands was produced by the primer SCoT 3 (19 bands), followed by SCoT 21 (18 bands), SCoT 11 (17 bands) and SCoT 13 (17 bands). Out of the 15 SCoT primers, SCoT 6 yielded the minimum number of bands (2 bands). Furthermore, the PIC value ranged between 0.78 for SCoT 6 and 0.20 for SCoT 36, with an average PIC value of 0.52 for all tested primers, confirming their high efficiency in detecting polymorphism among the studied genotypes. Additionally, the mean values for Effective Multiplex Ratio (EMR), Resolving Power (RP), and Marker Index (MI) were 18.5 (ranging between 11.1 and 24.4), 19.2 (ranging between 1.9 and 26.5), and 9.4 (ranging between 3.6 and 11.9), respectively, highlighting the strong discriminatory capacity of the selected SCoT markers. A representative DNA fingerprinting pattern generated with primer SCoT 25 is shown in Figure 2, while the patterns obtained with all primers are shown in Supplementary Figure S1.

The maximum number of identified alleles (Na) of 2.00 was observed with SCoT 6 and SCoT 21 primers, followed by 1.95 for SCoT 3 and 1.94 for SCoT 13, while the minimum Na of 1.54 was recorded with SCoT 36 (Table 4). The average Na of 1.81 was recorded for all SCoT primers used. Moreover, the maximum and minimum Shannon’s information index (I) of 0.69 and 0.24 were recorded for SCoT 6 and SCoT 36, respectively, and the average I for all SCoT primers was 0.40. The maximum gene diversity (H) of 0.50 was detected with SCoT 6, while the minimum was 0.15 with SCoT 36, and a mean gene diversity value of 0.26 was observed for all SCoT tested markers.

### 3.2. Genetic Diversity and Differentiation Among Rosa Accessions

Genetic diversity indices were calculated for all accessions, treating the unidentified *Rosa* form as a separate taxon (Table 5). Genetic diversity per population varied considerably. The highest number of alleles (Na) was observed in the improved Bulgarian population ‘Population 5’, P1–P9 (1.38), followed by five accessions of *R. gallica*, G1–G4, D (1.24), and the local and Romanian populations of *R. alba*, A1–A4 (1.06), etc. In contrast, the *R. centifolia*, C1–C2, displayed the lowest Na value of 0.69. A similar pattern was observed for the effective number of alleles (Ne) and Shannon’s information index (I). Similar to Na, these indices showed the highest levels in *R. damascena* ‘Population 5’, P1–P9 (1.35; 0.29, respectively), and the lowest in Bulgarian cultivars of *R. damascena* (1.06; 0.05) (Table 5). In addition, the highest expected heterozygosity (He) was detected in ‘Population 5’, P1–P9 (0.20); in contrast, the value was twice as low in *Rosa* sp., Kl1–Kl8 (0.10), and the lowest in cultivar ‘Svezhen’, SV1–SV2 (0.03).

The highest number of loci (TB = 206), including five SCoT-specific loci, was detected in *R. damascena* ‘Population 5’, confirming the strong genetic potential of this improved Bulgarian population. Among the remaining species, the total number of bands ranged from 143 in *R. centifolia* to 188 in *R. gallica*. A considerable number of species-specific loci were also identified in *R. alba* (6), followed by *R. gallica* (4) and *R. centifolia* (4), whereas only a single species-specific locus was detected in *Rosa* sp. Across the 15 SCoT markers, only two cultivar-specific loci were identified—one in ‘Raduga’ and one in ‘Yanina’. However, twenty genotype-specific loci were identified that distinguish the cultivars from one another and are also detectable in the genotypes of *R. damascena* ‘Population 5’ and *R. alba* (Supplementary Table S1). The percentage of polymorphic loci among the species ranged from 8.51% to 50.64%, with the highest value again recorded for ‘Population 5’, followed by *R. gallica*, *R. alba*, *Rosa* sp., and *R. centifolia* (Table 5). In the cultivars, the percentage of polymorphism ranged from 8.09% to 11.91%, with the lowest level observed in ‘Svezhen’ and the highest in ‘Yanina’. The cultivar ‘Iskra’ exhibited the lowest total number of loci (TB = 151), whereas the highest number was recorded in ‘Eleina’ (TB = 161) (Table 5). Nei’s genetic distances among the cultivars ranged from 0.0843 to 0.2501, with the greatest distance observed between ‘Yanina’ and ‘Svezhen’ (Supplementary Table S2), reflecting their reduced genetic similarity and the overall limited diversity within the cultivar group. Among all accessions, Nei’s genetic distances varied from 0.0843 to 0.4490 (P7 and A1, Supplementary Table S1).

We also conducted an analysis of molecular variance (AMOVA) to determine how much of the total genetic variation in *Rosa* accessions is distributed between different levels of the population structure (Table 6). As expected, most genetic variation (61%) was retained within the individuals in each *Rosa* accession (*p* < 0.001) compared to the variation among groups (39%) (*p* < 0.001). These results demonstrated that a higher number of genetic variations are present within the assessed groups compared to among the groups. The calculated *Φ*_PT_ (PhiPT) value was 0.388, indicating a high level of population differentiation.

### 3.3. Principal Coordinate Analysis (PCoA) and Genetic Structure

To assess the genetic relationships and population structure among the 38 *Rosa* accessions, UPGMA clustering, PCoA, and STRUCTURE analysis were applied. This multivariate approach was chosen to complement the results of the cluster analysis. In general, clustering has higher resolution for discriminating closely related populations, whereas the PCoA can provide an informative representation of the overall genetic relationships and distances among major groups.

**Figure 3:**
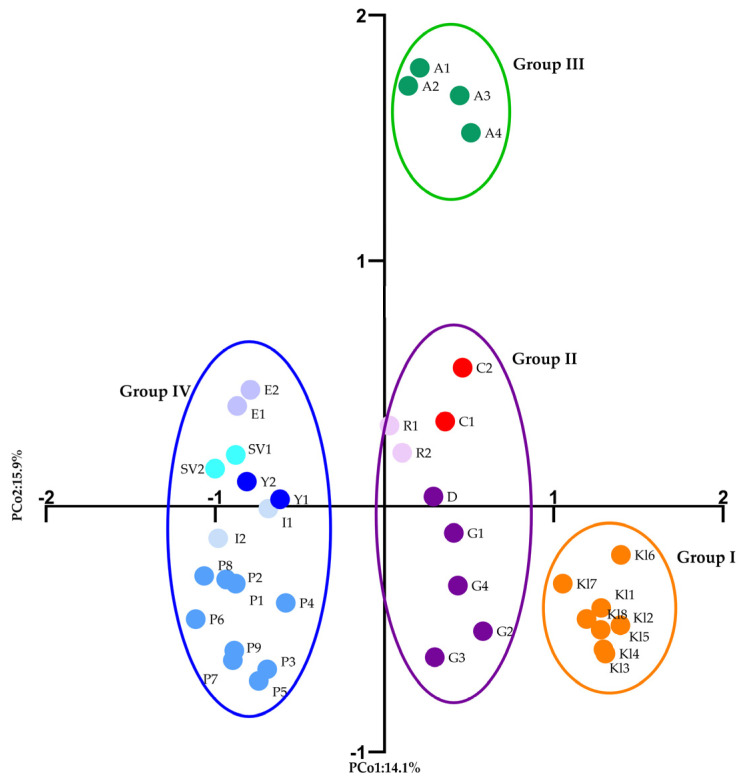
Principal coordinate analysis (PCoA) biplot of the studied 38 *Rosa* accessions. Kll–Kl8—*Rosa* sp., G1–G4, D—*Rosa gallica*, P1–P9—*Rosa damascena* ‘Population 5’, R1–R2—*Rosa* ‘Raduga’, C1–C2—*Rosa centifolia*, A1–A4—*Rosa alba*, *R. damascena* cultivars: I1–I2—‘Iskra’, Y1–Y2—‘Yanina’, E1–E2—‘Eleina’, SV1–SV2—‘Svezhen’.

Overall, the first three axes of the PCoA based on the genetic distances derived from the SCoT-PCR profiles of the fifteen markers explained 30% of the total variance: 14.19%, 8.74%, and 7.14%, respectively. The PCoA biplot (Figure 3) separated the 38 *Rosa* accessions into four major genetic groups: The first group includes the *Rosa* sp. accessions (Kl1-Kl8), which are classified as a single subpopulation, indicating that the genetic relationship between this population and the other populations is distant. The second group includes the five accessions of *R. gallica* (G1–G3, G4, D), as well as ’Raduga’ (R1–R2) and *R. centifolia* (C1–C2), indicating that their genetic relationship is the closest. Similar to Group I, all individuals of *R. alba* (A1–A3) in Group 3 formed a single cluster, demonstrating genetic distance from all other groups. Finally, Group 4 comprises all Bulgarian cultivars (E1–E2, I1–I2, SV1–SV2, and Y1–Y2) together with the Bulgarian population of *R. damascena*, ‘Population 5’ (P1–P9). The third PCoA axis reveals a clear separation of *R. centifolia* accessions (C1 and C2) from the remaining accessions in the second group, indicating their distant genetic profile compared to other rose species (Supplementary Figure S2).

**Figure 4:**
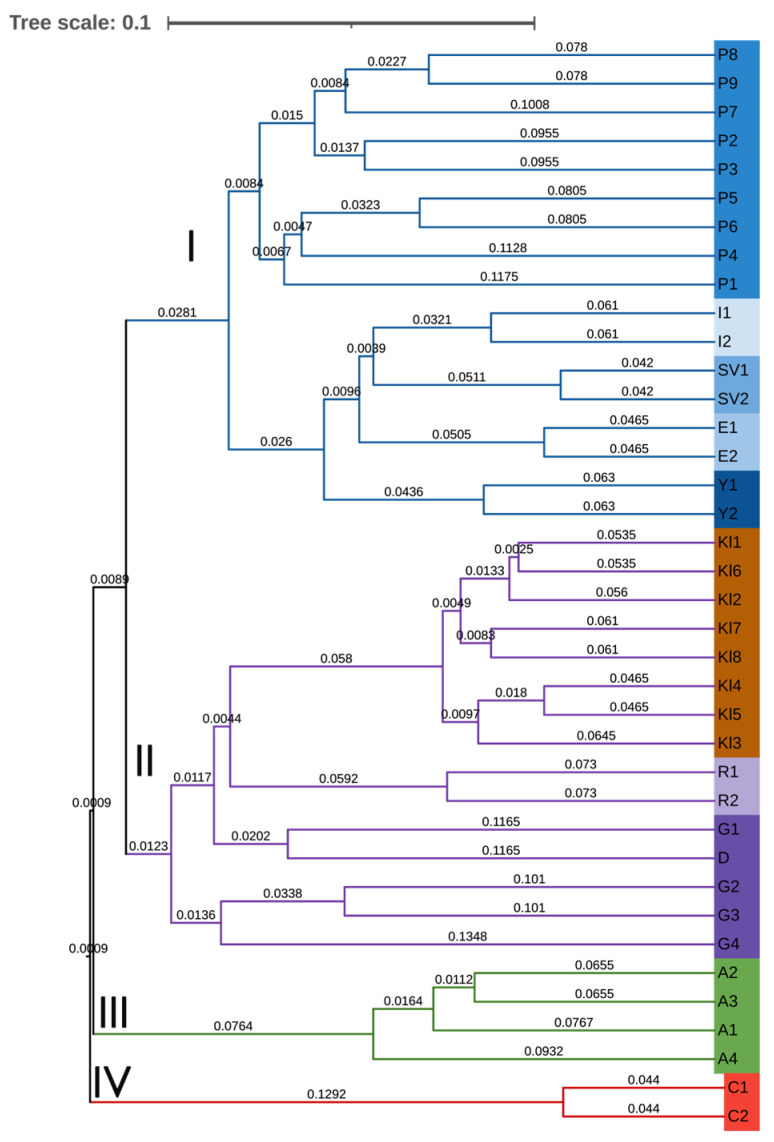
UPGMA-based dendrogram of 38 *Rosa* accessions based on molecular data from 15 SCoT markers Kll–Kl8—*Rosa* sp., G1–G4, D—*Rosa gallica*, P1–P9—*Rosa damascena* ‘Population 5’, R1–R2—*Rosa* ‘Raduga’, C1–C2—*Rosa centifolia*, A1–A4—*Rosa alba*, *R. damascena* cultivars: I1–I2—‘Iskra’, Y1–Y2—‘Yanina’, E1–E2—‘Eleina’, SV1–SV2—‘Svezhen’.

The subsequent cluster analysis based on Nei’s genetic distances confirmed the grouping obtained from the PCoA analysis, clearly separating *R. centifolia* and *R. alba* accessions into distinct clusters, again resolving four major clusters. Cluster 1 comprises *R. damascena* ‘Population 5’ together with all Bulgarian cultivars. Cluster 2 includes all accessions of *R. gallica* and all individuals of *Rosa* sp., arranged into separate subclusters, with the complex hybrid ‘Raduga’ positioned in an intermediate subcluster between them. *R. alba* and *R. centifolia* each form a separate, well-defined cluster (Figure 4). Within the *R. gallica* varietal group, accession G1, originating from IREMC, clusters together with the Romanian representative D, whereas G2 and G3, also germplasm maintained at IREMC, form a subcluster with the second Romanian accession, G4.

**Figure 5:**
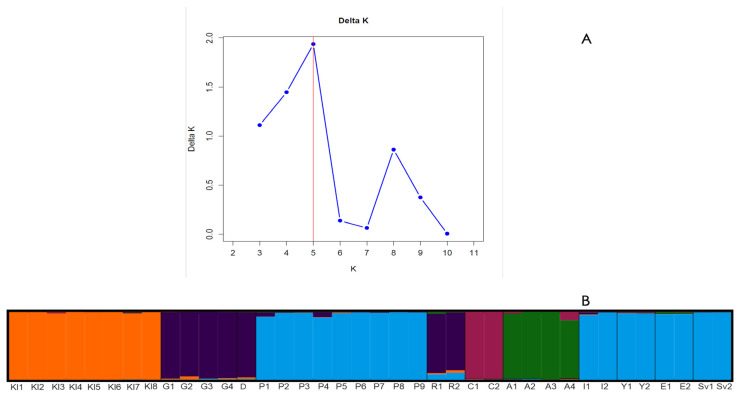
(**A**) Delta K plot from STRUCTURE analysis of 38 *Rosa* accessions based on SCoT-PCR amplification. The plot displays mean delta K values for 20 independent runs for each value of K (2–11). The highest value was at K = 5, indicating the presence of five distinct groups among the 38 *Rosa* accessions. (**B**) STRUCTURE bar plot showing the estimated membership of each accession in the five groups. Each vertical line represents the individuals, while different colours indicate the distinct groups. The length of the coloured segments shows the estimated proportion of membership in the corresponding genetic clusters. Kll–Kl8—*Rosa* sp., G1–G4, D—*Rosa gallica*, P1–P9—*Rosa damascena* ‘Population 5’, R1–R2—*Rosa* ‘Raduga’, C1–C2—*Rosa centifolia*, A1–A4—*Rosa alba*, *R. damascena* cultivars: I1–I2—‘Iskra’, Y1–Y2—‘Yanina’, E1–E2—‘Eleina’, SV1–SV2—‘Svezhen’.

The genetic structure analysis was performed, and ∆K was used to determine the optimal number of genetic clusters (K). The highest ∆K value was observed at K = 5 (Figure 5A). These results indicated that the 38 *Rosa* accessions can be assigned to five major groups based on their *Q* values, which reflect the proportional membership of each individual to a given ancestral cluster and are represented by different colours (Supplementary Table S3). Cluster Q1 contains the two accessions of *R. centifolia* (C1–C2). Cluster Q2 includes all four accessions of *R. alba* (A1–A4). Cluster Q3 comprises all individuals belonging to *R. damascena*—‘Population’ 5 (P1–P9)—as well as the Bulgarian cultivars ‘Eleina’ (E1–E2), ‘Yanina’ (Y1–Y2), ‘Iskra’ (I1–I2), and ‘Svezhen’ (SV1–SV2). Cluster Q4 includes all accessions of *R. gallica*: three accessions from IREMC (G1, G2–G3) and two accessions from Romania (G4 and D). Cluster Q5 encompasses all eight accessions of *Rosa* sp.

The two samples of ‘Raduga’ were classified as admixed genotypes due to their mixed ancestry components (*Q* value < 0.6). They showed predominant membership in clusters Q3 and Q4, with a minor proportion assigned to Q5 (Figure 5B, Supplementary Table S3).

## 4. Discussion

Molecular markers were extensively employed for the characterization of germplasm, analysis of genetic diversity, determination of origin, estimation of genetic distances, gene mapping, and marker-assisted selection [52,53,54,55,56,57]. Thus, numerous marker systems such as simple sequence repeats (SSR), randomly amplified polymorphic DNA (RAPD), inter-simple sequence repeats (ISSR), universal rice primers (URP), start codon-targeted primers (SCoT) and cis-element amplified polymorphism (CEAP) have been applied in studies of genetic diversity [15,19,33,58,59] and population structure [2,18,60] within the genus *Rosa*.

The present study investigates the application of gene-targeted SCoT markers for assessing genetic diversity and analysing genetic relationships among 38 *Rosa* accessions. The SCoT marker technique is simple, cost-effective, rapid, efficient, and highly reproducible, requiring only a small amount of DNA and no prior knowledge of DNA sequence information [32]. These markers were designed based on the ATG context, a conserved region flanking the translation initiation codon; consequently, SCoT markers are associated with functional genes and their correlated traits [32]. In comparison with RAPD, AFLP, and ISSR marker systems, SCoT represents a gene-targeted, multilocus approach capable of generating more biologically meaningful information and is particularly effective for detecting high levels of genetic polymorphism [55,61,62].

### 4.1. Efficiency of SCoT Markers and Overall Genetic Diversity

The fifteen SCoT markers employed in this study showed high informativeness and discriminatory power, as indicated by the mean values of polymorphic information content (PIC = 0.52), effective multiplex ratio (EMR = 18.5), marker index (MI = 9.4), and resolving power (RP = 19.2). These values revealed substantial genetic diversity among the 38 *Rosa* accessions (I = 0.40, He = 0.26) and a high level of polymorphism (89%). These findings demonstrate that, despite the traditionally assumed narrow genetic base of oil-bearing roses [14,15,63], gene-targeted markers can uncover considerable hidden variation. Previous studies have similarly demonstrated the effectiveness of SCoT markers, revealing high levels of genetic polymorphism in rose cultivars, and have highlighted their value for germplasm management, propagation strategies, and the conservation of genetic resources [40,60,64]. They have been used to detect interspecific variation [33], distinguish different cultivars [40,64], and identify populations [2,18,19,60]. The assessment of genetic diversity with SCoT markers in roses enables effective germplasm classification, guiding the selection of diverse parental lines for breeding programs [33].

### 4.2. Genetic Resources of Rosa damascena and Their Significance

The opportunities to improve industrial *R. damascena* cultivars are largely restricted to clonal selection, as maintaining the traditional aroma, key phenotypic traits, and the characteristic composition of rose oil is essential. Consequently, global rose oil production depends on one or only a few closely related genotypes [14]. However, molecular technologies can substantially accelerate the selection process compared to conventional approaches. They can also provide a means to meet the ongoing demand for novel cultivars by facilitating the identification of suitable parents or populations with desirable traits within the existing genetic resources of the Damask rose [14].

In the present study, we analyzed the genetic potential of 17 *R. damascena* accessions (nine from ‘Population 5’ and eight representing four cultivars), all maintained in the scientific experimental collection of IREMC. This germplasm represents the foundation of modern rose cultivation in Bulgaria, yet it remains insufficiently characterized at the molecular level. Earlier studies based on SSR markers reported a genetic uniformity among 24 accessions of clonal lines and cultivars of *R. damascena* from the IREMC collection [15]. A more recent study [19] using ISSR markers confirmed the limited genetic potential for selection within the Bulgarian *R. damascena* cultivars (I = 0.16, He = 0.10). However, the same study also revealed substantial genetic variability in the widely cultivated population of the Kazanlik rose (‘Population 5’), where a high polymorphism was detected among 12 accessions (I = 0.36, He = 0.24) [19]. In line with these earlier findings, the present study corroborates this trend by revealing a higher genetic diversity within the nine accessions from the IREMC ‘Population 5’ compared to the eight accessions representing the Bulgarian cultivars (I = 0.22, He = 0.15, Nei’s genetic distance = 0.0843–0.2501). The markedly higher genetic diversity (Ne = 1.35, I = 0.29, He = 0.20) detected within the nine accessions of the locally improved *R. damascena* ‘Population 5’ reflects the traits-based selection approach used for its development. Four distinct clones (‘Svezhen 188’, ‘Svezhen 189’, ‘Svezhen 191’ and ‘Svezhen 190’) were combined in the propagation of this population [38]. These results further highlight the importance of ‘Population 5’ as a key reservoir of genetic variation that can support future breeding and selection programs.

The genetic polymorphism among Bulgarian *R. damascena* accessions has also been reported in a study of 16 populations from Greece, Turkey, France, and Bulgaria [18]. In that study, three Bulgarian accessions originating from production fields were analyzed; one accession clustered with genotypes from Turkey and Greece, whereas the other two formed distinct clusters and exhibited greater genetic similarity to French genotypes [18]. The genetic diversity in *R. damascena* has also been documented in several regions worldwide, including Morocco (36 accessions evaluated using 13 ISSR markers [4]), India (29 accessions analyzed using 36 SCoT markers [40]), and Iran (40 accessions from five regions assessed with 12 SCoT primers and 14 URP primers [2]). Additionally, Chtourou [3] highlights the species’ strong adaptive capacity, noting that epigenetic mechanisms contribute to its adjustment to local factors such as climate and cultivation practices, resulting in both phenotypic and genetic modifications [3].

Among the four Bulgarian cultivars examined, the percentage of polymorphic loci ranged from 8.09% to 11.91%, with the lowest value observed in the clonally selected cultivar ‘Svezhen’ and the highest in the mutant-derived ‘Yanina’. Nei’s genetic distances among cultivars varied from 0.0843 to 0.2501, with the greatest divergence detected between ‘Yanina’ and ‘Svezhen’, reflecting their distinct breeding origins. The SCoT markers additionally identified 20 cultivar-specific loci that require further investigation. These loci may relate to specific agronomic traits or serve for cultivar identification and authentication, as shown in studies for other plant species [65,66,67,68]. Overall, our findings provide an evidence-based framework for developing marker-based selection strategies to improve the essential oil productivity in Bulgarian rose cultivars.

### 4.3. Genetic Position of the Unidentified Bulgarian Rose Genotype

Another major contribution of the present study is the molecular characterization of the unknown Bulgarian rose genotype (*Rosa* sp.) found in an old rose plantation in the nearby town of Klisura. All analytical approaches applied (PCoA, UPGMA, and STRUCTURE) consistently placed this genotype in a distinct genetic group, clearly separated from all studied *R. damascena* accessions. In the STRUCTURE analysis, the *Rosa* sp. genotype showed no evidence of gene flow from the genotypes included in the study. In the cluster analysis, however, *Rosa* sp. formed a separate sub-cluster most closely related to *Rosa* ‘Raduga’ and all *R. gallica* accessions, suggesting a closer relationship with *R. gallica* and a possible complex hybrid origin involving this species. All eight analyzed *Rosa* sp. samples exhibited low genetic diversity (Ne = 1.17, I = 0.15, He = 0.10), supporting the information provided by the owner that the plantation in Klisura originated from vegetative propagation of a single mother plant (personal communication). The low intra-group variability, along with the presence of only one genotype-specific locus, indicates a high degree of genetic uniformity, likely resulting from this vegetative propagation. Further studies using additional molecular, morphological and phytochemical analyses are needed to better clarify its taxonomic status, essential oil quality characteristics, and potential economic value. Additional rose species not included in the present study will contribute to analyzing the genetic relationships of *Rosa* sp. with other oil-bearing roses.

### 4.4. Genetic Relationships Among Oil-Bearing Rosa Species

Our interspecific analyses clearly discriminated the major taxa included in the study. All *R. damascena* accessions (including ‘Population 5’ and the cultivars) formed a stable and well-defined group distinct from *R. gallica*, *R. alba*, and *R. centifolia*. The *R. gallica* accessions (three from Bulgaria and two from Romania) clustered into a separate, internally structured group. This pattern indicates a high degree of genetic relatedness regardless of geographic origin and supports the hypothesis of long-term cultivation and exchange of this species within the region. It is widely considered that in roses, a primary factor contributing to the lack of strong correlation between genetic and geographic divergence is human intervention [69]. Despite this, the groups analyzed in the present study exhibited greater divergence at the intrargroup level (61%) than at the intergroup level (39%), a pattern also reported in other studies on roses [2,5]. *Rosa alba* and *R. centifolia* were resolved as distinct units, while the hybrid cultivar ‘Raduga’ exhibited an intermediate, admixed profile, confirming the sensitivity of the SCoT markers in detecting its complex hybrid origins. The high genetic differentiation observed in our study (*Φ*_PT_ = 0.388) is consistent with the hybrid origin and complex polyploid nature of these species. Similarly to *R. damascena*, a previous study using SSR markers and genetic materials from the IREMC experimental collection reported that *R. alba* production largely relies on a single genotype [63].

A recent SSR-based study of *R. gallica* accessions identified key cultivars from the *R. centifolia*, *R. alba*, and Damask groups as interspecific hybrids of *R. gallica*, but could not identify the other species involved in these hybridizations. The authors recommended using a specific set of markers to resolve intra-generic relationships in such cultivars to uncover the origins of *R. centifolia*, *R. alba*, and Damask roses [70]. Our SCoT-based study further separated these three groups in population structure and cluster analyses. However, the first two axes of the PCoA analysis (Figure 3) indicated similarity of *R. centifolia* accessions to *R. gallica* and the cultivar ‘Raduga’, which is known to be a complex hybrid of *R. gallica*. This was clearly reflected in the STRUCTURE analysis, showing the mixed origin for ‘Raduga’ (*Q* value < 0.6) and the resolution of the primers used. All analytical methods applied (Figure 3, Figure 4 and Figure 5 and Figure S2) successfully distinguished both *R. centifolia* accessions as separate genotypes. Overall, the AMOVA results revealed a higher proportion of the genetic variation within groups than among groups (61%), thus underlining the importance of individual-level diversity and intra-population variation for effective genetic resource management. This is particularly relevant for oil-bearing roses, where the genetic base is limited and selection pressure is high.

### 4.5. Genetic Distinctiveness, Possible Historical Origin, and Conservation Significance of the Studied Romanian Roses

The Romanian roses included in this study, two *R. gallica* and one *R. alba* accession from southern Romania, have likely been traditionally cultivated for household uses such as jam and herbal tea preparation. Clarifying their genetic relationships with modern cultivars, while conserving these genotypes, is an important aspect of rose research. Both species show high resistance to adverse environmental conditions and low cultivation requirements [71], characteristics confirmed in current studies by the strong vegetative propagation capacity of *R. alba* from Cărbunești (A4) and *R. gallica* from Poeni (G4).

Field observations further indicate that several rose plants from Cărbunești occur in multiple household gardens within the same locality, suggesting long-term vegetative propagation from closely related source plants. This pattern, together with their genetic distinctiveness and morphological characteristics, indicates that these accessions are unlikely to represent modern rose cultivars. The *R. gallica* accession from Poeni (G4) exhibits phenotypic features comparable to those described for historical cultivars with a *gallica* background, such as Marbled Rose (*Rosa marmorea*, sensu historical literature), which is currently reported mainly from botanical collections. Although direct genetic comparison with authenticated *R. marmorea* material is not yet available, the observed similarities point to a possible relationship with ancient rose lineages described in pre-modern horticultural literature.

It is known that *R. alba* thrives in cooler climates and on poor soils, suggesting favourable prospects for oil production. Therefore, it is suitable for cultivation in marginal or northern areas where it could support organic production and provide economic opportunities for disadvantaged communities [71]. Other benefits from rose cultivation could include landscape stabilization in erosion- and landslide-prone regions [71,72]. Given their cold tolerance, general hardiness, disease resistance, low maintenance needs, and self-supporting growth habit—especially in *R. gallica*—both species are well suited to low-input, sustainable agricultural systems, supporting their inclusion in genetic diversity assessments and future studies on essential oil production under diverse environmental conditions [73].

## 5. Conclusions

The present study demonstrates that start codon-targeted (SCoT) markers are powerful and reliable tools for discriminating among oil-bearing rose species, cultivars, and individual genotypes. The high level of polymorphism detected confirms their suitability for genetic characterization and population structure analysis within the genus of *Rosa*.

The locally improved *R. damascena* ‘Population 5’ exhibited the highest genetic diversity among the studied materials, demonstrating its importance as a key genetic source for future breeding and selection programs. In contrast, the Bulgarian *R. damascena* cultivars showed comparatively lower diversity, reflecting their clonal origin. The cultivar-specific SCoT loci identified in this study should be further examined for potential associations with agronomic traits and, in combination with the genetic distance estimates, may assist in informed parental selection.

The unidentified Bulgarian genotype (*Rosa* sp.) was clearly differentiated from *R. damascena* accessions, showing greater genetic similarity to *R. gallica* and the cultivar ‘Raduga’, suggesting a closer evolutionary relationship with *R. gallica*.

The unknown *Rosa* sp. from Bulgaria, along with the Romanian *R. gallica* and *R. alba* accessions, represents genetically distinct traditional germplasm, likely maintained through long-term vegetative propagation, and constitutes valuable resources for diversity conservation and sustainable rose breeding. Overall, this study extends our understanding of the genetic diversity and structure of oil-bearing roses and adds valuable information on the relationships among commonly used *Rosa* genotypes. The data obtained can support future breeding and conservation efforts aimed at enhancing productivity, adaptability, and sustainable management of this economically and culturally significant crop.

## Figures and Tables

**Table 1:** Details of *Rosa* accessions collections used in the study.

ID	Species	Characteristics	Source
Kl1	*Rosa* sp.	Shrub low-growing, to 100 cm tall; stems slender, erect. Prickles sparse to nearly absent, present only on juvenile shoots, caducous. Flowers double, strongly fragrant; petals magenta-pink, adaxially white-striated, abaxially pale pink. Plants producing abundant suckers, forming dense clumps; suitable for vegetative propagation. Resistant to rust, moderately resistant to black spot; not attacked by *Agrilus* spp.	IREMC
Kl2			IREMC
Kl3			IREMC
Kl4			IREMC
Kl5			IREMC
Kl6			IREMC
Kl7			IREMC
Kl8			IREMC
G1	*Rosa gallica*	Shrub up to 150 cm, with moderate prickle coverage. Leaflets 3–7. Flower fragrant, magenta pink, ~18 petals, with 150 bright yellow stamens.	IREMC
G2		Shrub bushy, luxuriant, with large prickles. Flowers double, small, scented, bright pink, 26 petals.	IREMC
G3			IREMC
G4		A tall, robust rose (up to 2 m) with abundant, once-per-year blooming, bearing large double purple-marbled flowers with a distinctive, strong fragrance. Easy to propagate, requires minimal care, with high productivity.	Romania
D		Non-remontant rose, bush very thorny, up to about 150 cm. Flowers globular, pompon-like about 7 cm in diameter, with a rich fuchsia colour, strongly fragrant, ~100 petals, blooming abundantly once in May–June with slightly extended flowering period. Susceptible to black spot.	Romania
P1	*Rosa damascena*	Improved local population of the Kazanlik oil-bearing rose ‘Population 5’, perennial shrub, 1.5–2 m tall, strongly branched, with numerous thorns. Flowers large, pink, strongly fragrant, 3–9 (or more) per branch, bloom sequentially.	IREMC
P2			IREMC
P3			IREMC
P4			IREMC
P5			IREMC
P6			IREMC
P7			IREMC
P8			IREMC
P9			IREMC
A1	*Rosa alba*	Clones from a diverse *R. alba* populations in Bulgaria differing in petal colour, petal number, stamen number, pistil type, and organoleptic traits (fragrance).	IREMC
A2			IREMC
A3			IREMC
A4		Shrub vigorous, peduncles glandular–hispid, calyx tube oval-fusiform. Flowers semi-double, strongly fragrant with light citrus notes; double white blooms (~50 petals) with pale pink centre at full anthesis, ~7 cm diameter, profusely flowering. Plant with long arching branches up to ~2.75 m; leaves grey-green, with seven serrated leaflets.	Romania
R1	*Rosa* ‘Raduga’	Shrubs compact, well-leafed, 100–120 cm tall, leaves compound, 5–7-foliolate, with numerous veins and small prickles along main veins. Flowers bright pink; hips large and abundant. Cultivar with high fruit productivity and notable rust resistance.	IREMC
R2			IREMC
C1	*Rosa centifolia*	Shrubs 150 cm tall; shoots slender, well-branched, arching during flowering. Stems with numerous prickles; leaves pinnate, composed of multiple leaflets. Flowers large, showy, few per inflorescence; plant characterized by high floral biomass yield.	IREMC
C2			IREMC
I1	*Rosa damascena*	Cultivar ‘Iskra’; morphologically similar to ‘Population 5’, but more vigorous, producing more numerous and longer lateral branches. Shoots bearing up to eight flowers; individual blossoms with up to 36 petals.	IREMC
I2			IREMC
Y1		Cultivar ‘Yanina’; morphologically similar to ‘Population 5’. Shrubs upright, robust, with enhanced cold tolerance and increased resistance to rust and black spot diseases.	IREMC
Y2			IREMC
E1		Cultivar ‘Eleina’; morphologically similar to ‘Population 5’. Shrubs up to 160 cm tall, stems upright and sturdy; flowering period extended. Plants with enhanced cold hardiness and increased resistance to leaf rust and black spot.	IREMC
E2			IREMC
SV1		Cultivar ‘Svezhen’; morphologically similar to ‘Population 5’. Produces a greater number of flower buds per flowering shoot.	IREMC
SV2			IREMC

**Table 2:** Sequence and annealing temperature of SCoT markers used in the genetic diversity analysis of 38 *Rosa* accessions.

Primer ID	Sequence	Annealing °C
SCoT 2	CAA CAA TGG CTA CCA CCC	55
SCoT 3	CAACAATGGCTACCACCG	55
SCoT 6	CAACAATGGCTACCACGC	50
SCoT 11	AAGCAATGGCTACCACCA	55
SCoT 12	ACGACATGGCGACCAACG	55
SCoT 13	ACGACATGGCGACCATCG	55
SCoT 15	ACGACATGGCGACCGCGA	55
SCoT 17	ACCATGGCTACCACCGAG	54
SCoT 19	ACCATGGCTACCACCGGC	58
SCoT 21	ACGACATGGCGACCCACA	55
SCoT 22	AACCATGGCTACCACCAC	55
SCoT 25	ACCATGGCTACCACCGGG	55
SCoT 31	CCATGGCTACCACCGCCT	57
SCoT 33	CCATGGCTACCACCGCAG	55
SCoT 36	GCAACAATGGCTACCACC	55

**Table 3:** PCR amplification results with 15 SCoT Primers: polymorphic bands (PB), monomorphic bands (MB), effective multiplex ratio (EMR), resolving power (RP), marker index (MI), polymorphic information content (PIC).

Primer ID	PB	MB	EMR	RP	MI	PIC	PB%
SCoT 2	12	3	19.60	19.3	9.25	0.47	80.0
SCoT 3	19	1	23.90	25.2	11.90	0.50	95.0
SCoT 6	2	0	18.00	1.9	14.00	0.78	100
SCoT 11	17	1	19.30	19.3	11.50	0.60	94.4
SCoT 12	15	4	20.90	26.5	8.60	0.41	78.9
SCoT 13	17	1	24.40	24.5	11.10	0.48	94.4
SCoT 15	11	4	19.20	20.7	7.30	0.38	73.3
SCoT 17	10	4	11.10	10.6	7.90	0.72	71.4
SCoT 19	16	3	19.20	22.8	10.20	0.53	84.2
SCoT 21	18	0	15.70	14.9	11.80	0.75	100
SCoT 22	7	3	17.64	13.3	7.90	0.45	70.0
SCoT 25	16	5	15.70	22.7	9.20	0.59	76.2
SCoT 31	13	4	17.6	20.6	8.80	0.50	76.5
SCoT 33	14	5	17.5	23.8	8.50	0.48	73.7
SCoT 36	7	6	17.9	22.8	3.60	0.20	53.8
Mean			18.5	19.2	9.4	0.52	89
Total	194	44					

**Table 4:** Genetic diversity of SCoT primers used: Total number of different (Na) and effective (Ne) alleles, gene diversity (H), Shannon’s information index (I).

Primer ID	Na	Ne	H	I
SCoT 2	1.80	1.30	0.24	0.37
SCoT 3	1.95	1.56	0.32	0.48
SCoT 6	2.00	1.99	0.50	0.69
SCoT 11	1.88	1.37	0.26	0.39
SCoT 12	1.79	1.33	0.21	0.34
SCoT 13	1.94	1.53	0.31	0.64
SCoT 15	1.73	1.21	0.14	0.30
SCoT 17	1.77	1.40	0.25	0.38
SCoT 19	1.80	1.43	0.26	0.41
SCoT 21	2.00	1.52	0.33	0.49
SCoT 22	1.70	1.38	0.23	0.34
SCoT 25	1.76	1.45	0.26	0.39
SCoT 31	1.76	1.34	0.21	0.33
SCoT 33	1.73	1.14	0.22	0.34
SCoT 36	1.54	1.29	0.15	0.24
Mean	1.81	1.42	0.26	0.40

**Table 5:** Genetic diversity estimates in *Rosa* accessions: Kl—*Rosa* sp., G, D—*Rosa gallica*, P—*Rosa damascena* ‘Population 5’, C—*Rosa centifolia*, A—*Rosa alba*, R—*Rosa* ‘Raduga’, *R. damascena* cultivars: I—‘Iskra’, Y—‘Yanina’, E—‘Eleina’, SV—‘Svezhen’. Total bands (TB); Private bands (Pb); Percentage of polymorphic loci (PB%); Number of individual (N), different (Na) and effective (Ne) alleles; Shannon’s information index (I); Expected heterozygosity (He).

ID	TB	Pb	PB%	N	Na	Ne	I	He
Kl1–Kl8	170	1	28.09	8	1.01	1.17	0.15	0.10
G1–G4, D	188	4	43.83	5	1.24	1.27	0.24	0.16
P1–P9	206	5	50.64	9	1.38	1.35	0.29	0.20
A1–A4	182	6	28.09	4	1.06	1.21	0.17	0.12
C1–C2	143	4	8.51	2	0.69	1.06	0.05	0.04
R1–R2	161	1	11.06	2	0.80	1.07	0.07	0.05
I1–I2	151	0	11.49	2	0.76	1.08	0.07	0.05
Y1–2	157	1	11.91	2	0.79	1.08	0.07	0.05
E1–E2	161	0	8.94	2	0.77	1.06	0.05	0.04
SV1–SV2	152	0	8.09	2	0.73	1.06	0.05	0.03
Mean	167.1	2.2	21.1	3.8	0.92	1.14	0.12	0.08

**Table 6:** Analysis of molecular variance (AMOVA) of studied *Rosa* accessions.

Source	df	SS	MS	Est. Var.	% Variation	*p* Value
Among Groups	9	595.699	66.189	12,759	39%	<0.001
Within Groups	28	564.275	20.153	20.153	61%	<0.001
Total	37	1159.974		32.911	100%	

## Data Availability

The original contributions presented in this study are included in the article. Further inquiries can be directed at the corresponding author.

## Supplementary Materials

The following supporting information can be downloaded at https://www.mdpi.com/article/10.3390/plants15050761/s1: Figure S1: Molecular profiles of 38 *Rosa* accessions in 15 SCoT primers (Subfigures 1~15); Figure S2: Principal coordinate analysis (PCoA) biplot of the 38 *Rosa* accessions studied; Table S1: Genotype-specific loci identified in Bulgarian cultivars of *Rosa damascena*; Table S2: Nei genetic distance and similarity; Table S3: *Q* value compositions of 38 *Rosa* accessions.

## References

[1] Smulders M.J., Arens P., Bourke P.M., Debener T., Linde M., De Riek J., Leus L., Ruttink T., Baudino S., Hibrant Saint-Oyant L. (2019). In the name of the rose: A roadmap for rose research in the genome era. Hortic. Res..

[2] Mostafavi A.S., Omidi M., Azizinezhad R., Etminan A., Badi H.N. (2021). Genetic diversity analysis in a mini core collection of Damask rose (*Rosa damascena* Mill.) germplasm from Iran using URP and SCoT markers. J. Genet. Eng. Biotechnol..

[3] Chtourou K., Salazar J.A., Ortuño-Hernández G., Mezghani N., Trifi-Farah N., Martínez-Gómez P., Krichen L. (2024). Genetic diversity and relationships among Tunisian wild and cultivated *Rosa* L. species. Plants.

[4] Lebkiri N., Abbas Y., Iraqi D., Gaboun F., Saghir K., Fokar M., Diria G. (2024). Morphological characterization and genetic diversity of a mini core collection of *Rosa damascena* from Morocco. J. Genet. Eng. Biotechnol..

[5] Saghir K., Abdelwahd R., Iraqi D., Lebkiri N., Gaboun F., El Goumi Y., Diria G. (2022). Assessment of genetic diversity among wild roses in Morocco using ISSR and DAMD markers. J. Genet. Eng. Biotechnol..

[6] Koopman W.J., Wissemann V., De Cock K., Van Huylenbroeck J., De Riek J., Sabatino G.J., Smulders M.J. (2008). AFLP markers as a tool to reconstruct complex relationships: A case study in *Rosa* (Rosaceae). Am. J. Bot..

[7] Harmon D.D., Chen H., Byrne D., Liu W., Ranney T.G. (2023). Cytogenetics, ploidy, and genome sizes of rose (*Rosa* spp.) cultivars and breeding lines. Ornam. Plant Res..

[8] Babaei A., Tabaei-Aghdaei S.R., Khosh-Khui M., Moradi H., Naghavi M.R., Kalantar E. (2007). Microsatellite analysis of Damask rose (*Rosa damascena* Mill.) accessions from various regions in Iran reveals multiple genotypes. BMC Plant Biol..

[9] De Cock K. (2008). Genetic Diversity of Wild Roses (*Rosa* spp.) in Europe, with an In-Depth Morphological Study of Flemish Populations. Ph.D. Thesis.

[10] Gaurav A.K., Namita, Raju D.V.S., Ramkumar M.K., Singh M.K., Singh B., Sevanthi A.M. (2022). Genetic diversity analysis of wild and cultivated *Rosa* species of India using microsatellite markers and their comparison with morphology-based diversity. J. Plant Biochem. Biotechnol..

[11] Salcă Roman G.M., Lehel L., Somsai A.P., Stoian-Dod R.L., Dan C., Bunea C.I., Sestras R.E. (2024). The use of genetic resources in rose breeding and creation of new rose cultivars through hybridization and selection. Not. Bot. Horti Agrobo..

[12] Ilieva Y., Dimitrova L., Georgieva A., Vilhelmova-Ilieva N., Zaharieva M.M., Kokanova-Nedialkova Z., Mileva M. (2022). In vitro study of the biological potential of wastewater obtained after the distillation of four Bulgarian oil-bearing roses. Plants.

[13] El Malahi S., Ganoudi M., Hassani L.M.I. (2025). Beyond beauty: A look at the Damask rose’s origin, history and geographical spread. Int. J. Agric. Environ. Res..

[14] Venkatesha K.T., Gupta A., Rai A.N., Jambhulkar S.J., Bisht R., Padalia R.C. (2022). Recent developments, challenges, and opportunities in genetic improvement of essential oil-bearing rose (*Rosa damascena*): A review. Ind. Crops Prod..

[15] Rusanov K., Kovacheva N., Vosman B., Zhang L., Rajapakse S., Atanassov A., Atanassov I. (2005). Microsatellite analysis of *Rosa damascena* Mill. accessions reveals genetic similarity between genotypes used for rose oil production and old Damask rose varieties. Theor. Appl. Genet..

[16] Pirseyedi S.M., Mardi M., Davazdahemami S., Kermani M.J., Mohammadi S.A. (2005). Analysis of the genetic diversity of Iranian Damask rose (*Rosa damascena* Mill.) genotypes using AFLP markers. Iran. J. Biotechnol..

[17] Farooq A., Kiani M., Khan M.A., Riaz A., Khan A.A., Anderson N., Byrne D.H. (2013). Microsatellite analysis of *Rosa damascena* from Pakistan and Iran. Hortic. Environ. Biotechnol..

[18] Ziogou F.-T., Kotoula A.-A., Hatzilazarou S., Papadakis E.-N., Avramis P.-G., Economou A., Kostas S. (2023). Genetic assessment, propagation and chemical analysis of flowers of *Rosa damascena* Mill. genotypes cultivated in Greece. Horticulturae.

[19] Zhelyazkova M., Grozeva N., Todorova M., Dobreva A., Badzhelova V., Georgieva S., Hristov P., Lazarova S. (2024). Assessment of genetic diversity of oil-bearing rose (*Rosa damascena* Mill.) using ISSR markers. Bulg. J. Agric. Sci..

[20] Reynders-Aloisi S., Sciberras E., Tarbouriech M.F. (2000). *Rosa gallica*: The French rose—Its natural diversity within and between wild populations in south-eastern France. XIX International Symposium on Improvement of Ornamental Plants.

[21] Monder M.J. (2014). Evaluation of growth and flowering of historical cultivars of *Rosa gallica* L.. Acta Agrobot..

[22] Gudin S. (2017). Overview of plant breeding. Hortic. Rev..

[23] Vukosavljev M., Zhang J., Esselink G.D., Van ’t Westende W.P.C., Cox P., Visser R.G.F., Arens P., Smulders M.J.M. (2013). Genetic diversity and differentiation in roses: A garden rose perspective. Sci. Hortic..

[24] Dobreva A., Nedeva D., Mileva M. (2023). Comparative study of the yield and chemical profile of rose oils and hydrosols obtained by industrial plantations of oil-bearing roses in Bulgaria. Resources.

[25] Gateva S., Jovtchev G., Angelova T., Gerasimova T., Dobreva A., Mileva M. (2024). Genotoxic and Anti-Genotoxic Potential of Hydrosols from Water–Steam Distillation of Oil-Bearing Roses *Rosa centifolia* L. and *Rosa gallica* L. from Bulgaria. Pharmaceuticals.

[26] Verma A., Srivastava R., Sonar P.K., Yadav R. (2020). Traditional, phytochemical, and biological aspects of *Rosa alba* L.: A systematic review. Future J. Pharm. Sci..

[27] Qi W., Chen X., Fang P., Shi S., Li J., Liu X., Zhang Z. (2018). Genomic and transcriptomic sequencing of *Rosa hybrida* provides microsatellite markers for breeding and taxonomy studies. BMC Plant Biol..

[28] Heo M.S., Han K., Kwon J.K., Kang B.C. (2017). Development of SNP markers using genotyping-by-sequencing for cultivar identification in rose (*Rosa hybrida*). Hortic. Environ. Biotechnol..

[29] Omidi M., Khandan-Mirkohi A., Kafi M., Rasouli O., Shaghaghi A., Kiani M., Zamani Z. (2022). Comparative study of phytochemical profiles and morphological properties of some Damask roses from Iran. Chem. Biol. Technol. Agric..

[30] Rusanov K., Kovacheva N., Atanassov A., Atanassov I. (2009). *Rosa damascena* Mill., the oil-bearing Damask rose: Genetic resources, diversity and perspectives for molecular breeding. Floric. Ornam. Biotechnol..

[31] Slavova G., Stefanova S. (2020). Production of roses and rose oil in Bulgaria for the period 2014–2018. Manag. Sustain. Dev..

[32] Collard B.C., Mackill D.J. (2009). Start codon targeted (SCoT) polymorphism: A novel DNA marker technique. Plant Mol. Biol. Rep..

[33] Hârţa M., Cornea-Cipcigan M., Pui D.A., Roman G., Cordea M.I. (2023). Characterization and identification of genetic diversity among rose genotypes using morphological and molecular markers. Sci. Pap. Ser. B Hortic..

[34] Chettri K., Majumder J., Mahanta M., Mitra M., Gantait S. (2024). Genetic diversity analysis and molecular characterization of tropical rose (*Rosa* spp.) varieties. Sci. Hortic..

[35] Salama A.M., Osman E.A., El-Tantawy A.A. (2019). Taxonomical studies on four *Mentha* species grown in Egypt through morpho-anatomical characters and SCoT genetic markers. Plant Arch..

[36] Liu S., Wang Y., Song Y., Khayatnezhad M., Minaeifar A.A. (2021). Genetic variations and interspecific relationships in *Salvia* (Lamiaceae) using SCoT molecular markers. Caryologia.

[37] Zhelyazkova M., Badzhelova V., Stanev S. (2025). Assessment of Genetic Diversity Among Bulgarian Lavender Varieties Using Scot Markers. Agronomy.

[38] Astadjov N. (1988). Study on the Diversity of the Kazanlak Rose Population and Selected Aspects of Propagation and Cultivation with a View to Improving Diversity in Bulgaria. Ph.D. Thesis.

[39] Nedkov N., Kanev K., Kovacheva N., Stanev S., Dzhurmanski A., Seikova K., Lambev H., Dobreva A. (2005). Handbook of the Main Essential Oil and Medicinal Crops.

[40] Agarwal A., Gupta V., Haq S.U., Jatav P.K., Kothari S.L., Kachhwaha S. (2019). Assessment of genetic diversity in 29 rose germplasms using SCoT marker. J. King Saud Univ. Sci..

[41] Roldan-Ruiz I., Dendauw J., Vanbockstaele E., Depicker A., De Loose M. (2000). AFLP markers reveal high polymorphic rates in ryegrasses (*Lolium* spp.). Mol. Breed..

[42] Nagaraju J., Damodar R.K., Nagaraja G.M., Sethuraman B.N. (2001). Comparison of multilocus RFLPs and PCR-based marker systems for genetic analysis of the silkworm, Bombyx mori. Heredity.

[43] Varshney R.K., Chabane K., Hendre P.S., Aggarwal R.K., Graner A. (2007). Comparative assessment of EST-SSR, EST-SNP and AFLP markers for evaluation of genetic diversity and conservation of genetic resources using wild, cultivated and elite barleys. Plant Sci..

[44] Prevost A., Wilkinson M.J. (1999). A new system of comparing PCR primers applied to ISSR fingerprinting of potato cultivars. Theor. Appl. Genet..

[45] Nei M. (1973). Analysis of gene diversity in subdivided populations. Proc. Natl. Acad. Sci. USA.

[46] Peakall R., Smouse P.E. (2006). GENALEX 6: Genetic analysis in Excel. Population genetic software for teaching and research. Mol. Ecol. Resour..

[47] Evanno G., Regnaut S., Goudet J. (2005). Detecting the number of clusters of individuals using the software structure: A simulation study. Mol. Ecol..

[48] Pritchard J.K., Stephens M., Donnelly P. (2000). Inference of population structure using multilocus genotype data. Genetics.

[49] Li Y.L., Liu J.X. (2018). StructureSelector: A web-based software to select and visualize the optimal number of clusters using multiple methods. Mol. Ecol. Resour..

[50] Kumar S., Stecher G., Suleski M., Sanderford M., Sharma S., Tamura K. (2024). MEGA12: Molecular Evolutionary Genetic Analysis version 12 for adaptive and green computing. Mol. Biol. Evol..

[51] Letunic I., Bork P. (2024). Interactive Tree of Life (iTOL) v6: Recent updates to the phylogenetic tree display and annotation tool. Nucleic Acids Res..

[52] Bidyananda N., Jamir I., Nowakowska K., Varte V., Vendrame W.A., Devi R.S., Nongdam P. (2024). Plant genetic diversity studies: Insights from DNA marker analyses. Int. J. Plant Biol..

[53] Sinha S., Singh D. (2022). Role of molecular markers for genetic diversity analysis in floricultural crops–A review. J. Ornam. Hortic..

[54] Pan H., Deng L., Zhu K., Shi D., Wang F., Cui G. (2024). Evaluation of genetic diversity and population structure of Annamocarya sinensis using SCoT markers. PLoS ONE.

[55] Hromadová Z., Gálová Z., Mikolášová L., Balážová Ž., Vivodík M., Chňapek M. (2023). Efficiency of RAPD and SCoT markers in the genetic diversity assessment of the common bean. Plants.

[56] Wang F., Chen X., Huang Z., Wei L., Wang J., Wen S., Liu Y., Zhou Y. (2025). Phenotypic Characterization and Marker–Trait Association Analysis Using SCoT Markers in Chrysanthemum (*Chrysanthemum morifolium* Ramat.) Germplasm. Genes.

[57] Al-Khayri J.M., Mahdy E.M.B., Taha H.S.A., Eldomiaty A.S., Abd-Elfattah M.A., Latef A.A.H.A. (2022). Genetic and morphological diversity assessment of five kalanchoe genotypes by SCoT, ISSR and RAPD-PCR markers. Plants.

[58] Visalakshi M., Muthulakshmi R., Ganga M., Boopathi N.M., Vasanth S., Ganesh S. (2023). Assessment of genetic diversity among the elite Rose (*Rosa* spp.) accessions using RAPD markers. Int. J. Environ. Clim. Change.

[59] Zhelyazkova M., Badzhelova V., Georgieva S., Lozanova L., Hristov P., Lazarova S. (2024). Genetic diversity assessment of selected rose genotypes using CEAP markers. Bulg. J. Agric. Sci..

[60] Boutsika A., Mellidou I., Grigoriadou K., Papapanastasi K., Krigas N., Maloupa E., Ganopoulos I., Xanthopoulou A. (2025). Molecular profiling of Greek native germplasm collection of *Rosa canina* L. for enhanced fruit extract production: A comprehensive approach utilizing neutral, gene, and exon-based markers. Genet. Resour. Crop Evol..

[61] Rai M.K. (2023). Start codon targeted (SCoT) polymorphism marker in plant genome analysis: Current status and prospects. Planta.

[62] Gogoi B., Wann S.B., Saikia S.P. (2020). Comparative assessment of ISSR, RAPD, and SCoT markers for genetic diversity in *Clerodendrum* species of North East India. Mol. Biol. Rep..

[63] Rusanov K., Kovacheva N., Rusanova M., Atanassov I. (2013). Flower phenotype variation, essential oil variation and genetic diversity among *Rosa alba* L. accessions used for rose oil production in Bulgaria. Sci. Hortic..

[64] Saidi A., Eghbalnegad Y., Hajibarat Z. (2017). Study of genetic diversity in local rose varieties (*Rosa* spp.) using molecular markers. Banat. J. Biotechnol..

[65] Feng S., Zhu Y., Yu C., Jiao K., Jiang M., Lu J., Shen C., Ying Q., Wang H. (2018). Development of species-specific SCAR markers, based on a SCoT analysis, to authenticate *Physalis* (Solanaceae) species. Front. Genet..

[66] Xu Y.X., Shen S.Y., Chen W., Chen L. (2019). Analysis of genetic diversity and development of a SCAR marker for green tea (*Camellia sinensis*) cultivars in Zhejiang Province. Biochem. Genet..

[67] Rahimi M., Nazari L., Kordrostami M., Safari P. (2018). SCoT marker diversity among Iranian *Plantago* ecotypes and their possible association with agronomic traits. Sci. Hortic..

[68] Karagöz H., Hosseinpour A., Karagöz F.P., Cakmakci R., Haliloglu K. (2022). Dissection of genetic diversity and population structure in oregano based on SCoT markers. Biologia.

[69] Reichel K., Herklotz V., Smolka A., Nybom H., Kellner A., De Riek J., Smulders M.J.M., Wissemann V., Ritz C.M. (2023). Untangling the hedge: Genetic diversity in European wild roses, *Rosa* L.. PLoS ONE.

[70] Pawula C. (2023). *Rosa gallica* L. and Other Gallic roses: Origin(s) and Role in the Genesis of Cultivated Roses. Ph.D. Thesis.

[71] Barbu F. (2022). Biodiversity of Species and Varieties of Roses Used in the Food and Cosmetic Industry. Master’s Thesis.

[72] Royal Horticultural Society. https://www.rhs.org.uk/.

[73] Dobreva A., Gerdzhikova M. (2013). Content and composition of the essential oil of *Rosa alba* L. during flower development. Agric. Sci. Technol..

