# Supplementary material for: Assessment of Genetic Diversity and Population Structure in Oil-Bearing Rose Genotypes Using Start Codon-Targeted (SCoT) Markers
Source: Plants (Basel). 2026 Mar 1;15(5):761. doi: 10.3390/plants15050761 (PMC12986590; doi:10.3390/plants15050761)
Supplement: Supplementary file 1 [file plants-15-00761-s001.zip › Figure S1. Molecular profiles of 38 Rosa accessions in 15 SCoT primers.pdf]

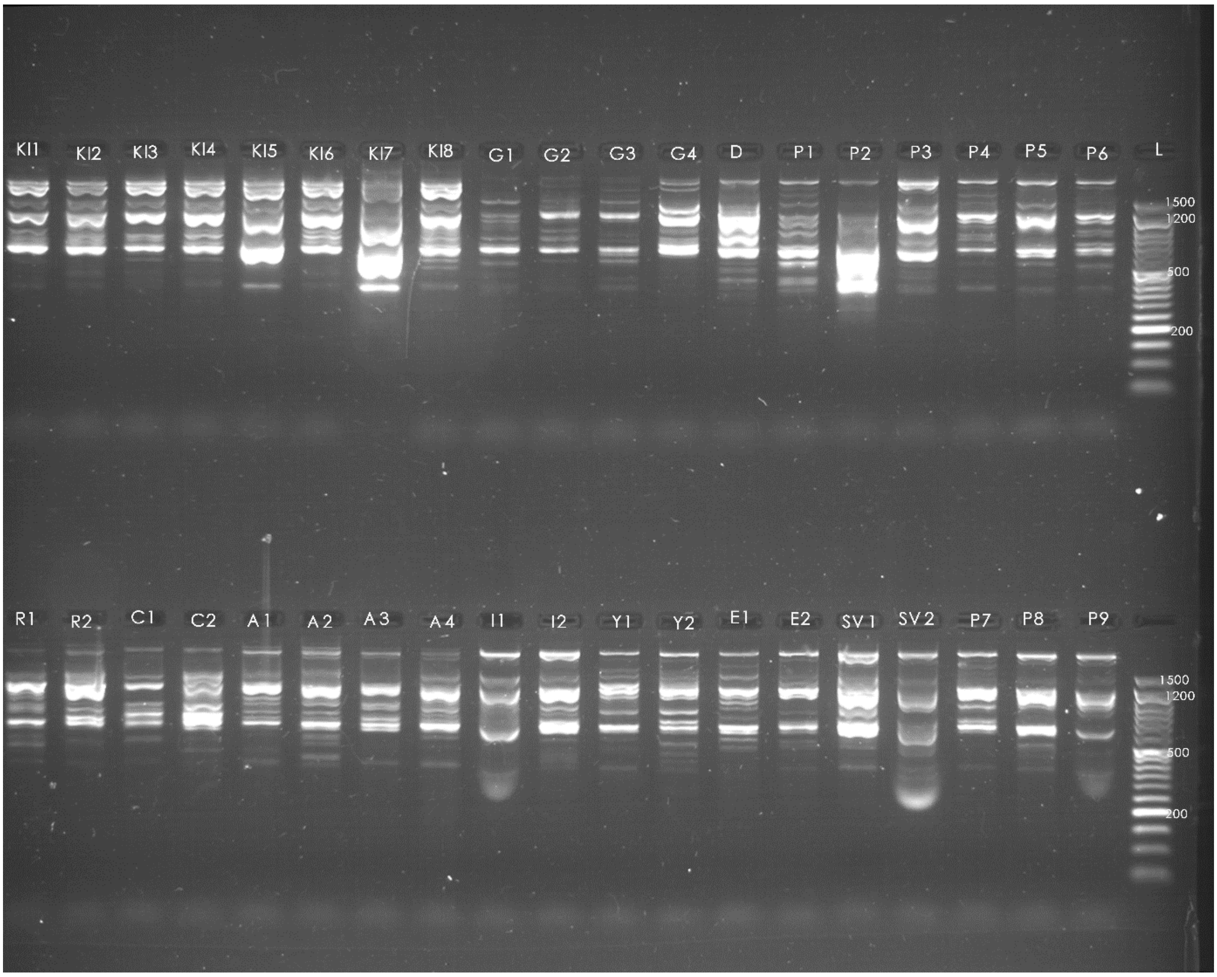

Subfigure 1. Molecular profiles of 38 *Rosa* accessions in SCoT 2 primer. Lanes marked as 1 to 38 which represent the accessions according to serial numbers in Kll - Kl8 – *Rosa* sp., G1 - G4, D – *R. gallica*, P1 - P9 – *R. damascena* 'Population 5', R1 - R2 – *Rosa* 'Raduga', C1 - C2 – *R. centifolia,* A1 - A4 – *R. alba*, *R. damascena* cultivars: I1 - I2 – 'Iskra', Y1 - Y2 – 'Yanina', E1 - E2 – 'Eleina', SV1 - SV2 – 'Svezhen'; L – Ladder 50 – 1500 bp

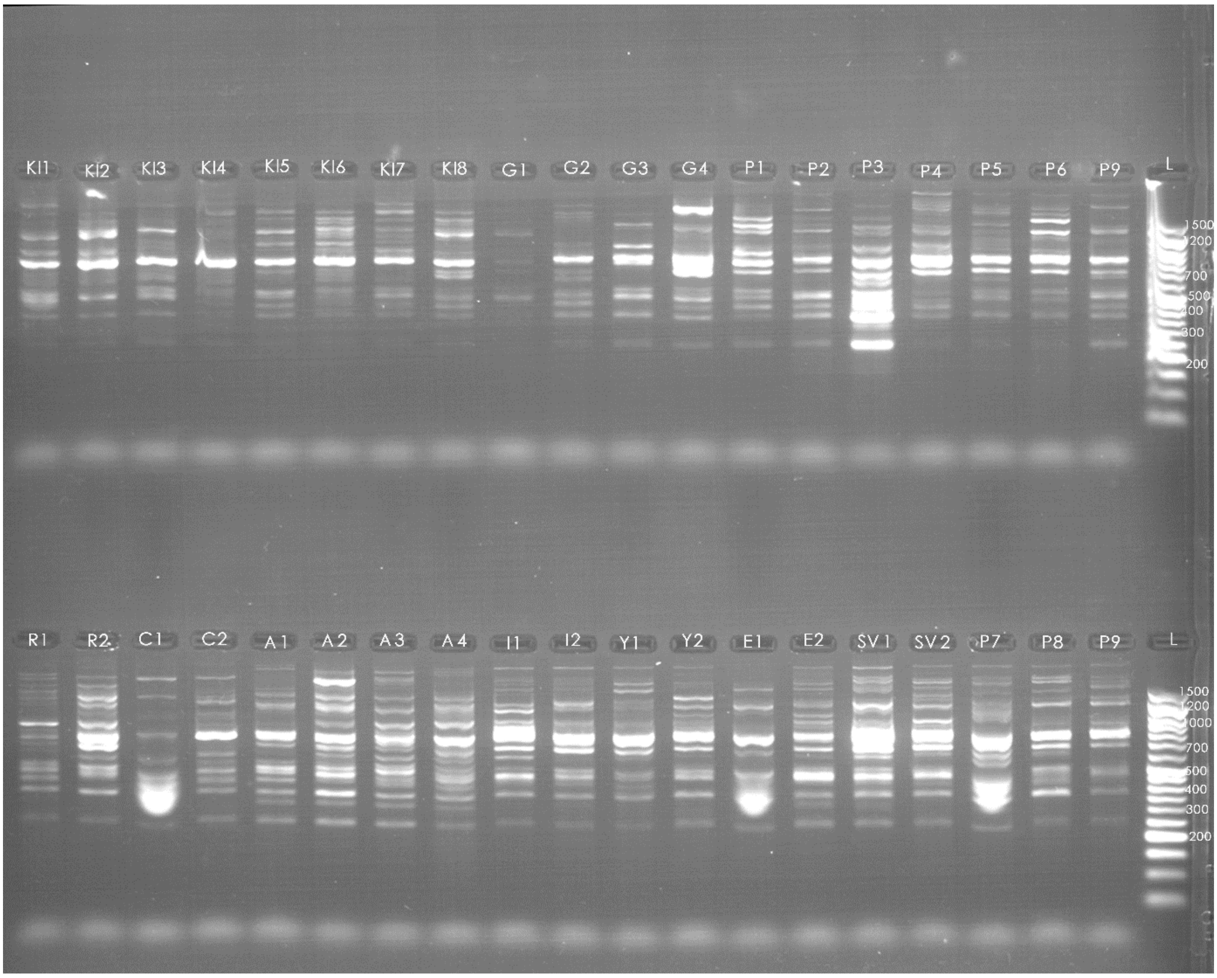

Subfigure 2. Molecular profiles of 38 *Rosa* accessions in SCoT 3 primer. Lanes marked as 1 to 38 which represent the accessions according to serial numbers in Kll - Kl8 – *Rosa* sp., G1 - G4, D – *R. gallica*, P1 - P9 – *R. damascena* 'Population 5', R1 - R2 – *Rosa* 'Raduga', C1 - C2 – *R. centifolia*, A1 - A4 – *R. alba*, *R. damascena* cultivars: I1 - I2 – 'Iskra', Y1 - Y2 – 'Yanina', E1 - E2 – 'Eleina', SV1 - SV2 – 'Svezhen'; L – Ladder 50 – 1500 bp

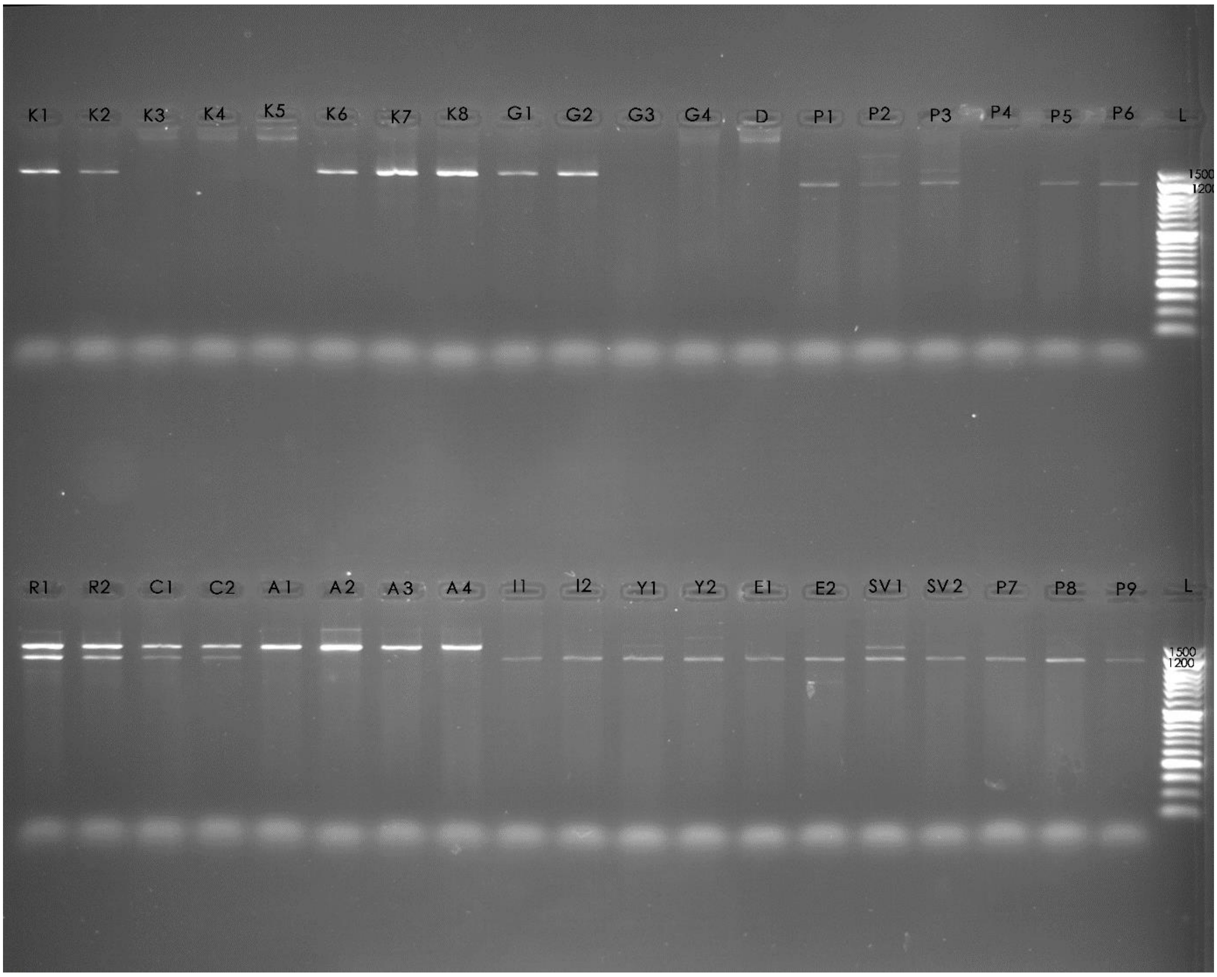

Subfigure 3. Molecular profiles of 38 *Rosa* accessions in SCoT 6 primer. Lanes marked as 1 to 38 which represent the accessions according to serial numbers in Kl1 - Kl8 – *Rosa* sp., G1 - G4, D – *R. gallica*, P1 - P9 – *R. damascena* 'Population 5', R1 - R2 – *Rosa* 'Raduga', C1 - C2 – *R. centifolia*, A1 - A4 – *R. alba*, *R. damascena* cultivars: I1 - I2 – 'Iskra', Y1 - Y2 – 'Yanina', E1 - E2 – 'Eleina', SV1 - SV2 – 'Svezhen'; L – Ladder 50 – 1500 bp

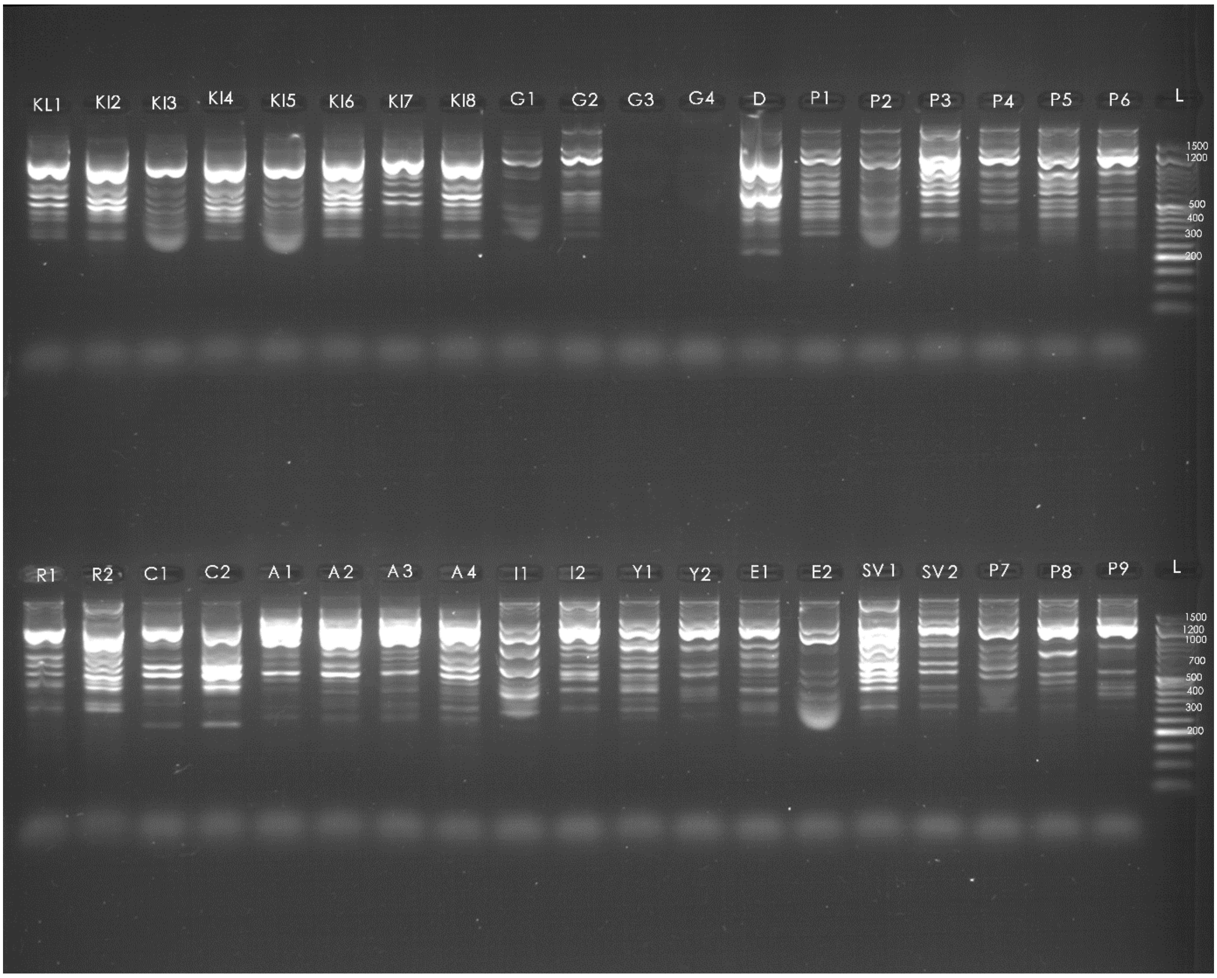

Subfigure 4. Molecular profiles of 38 *Rosa* accessions in SCoT 11 primer. Lanes marked as 1 to 38 which represent the accessions according to serial numbers in Kl1 - Kl8 – *Rosa* sp., G1 - G4, D – *R. gallica*, P1 - P9 – *R. damascena* 'Population 5', R1 - R2 – *Rosa* 'Raduga', C1 - C2 – *R. centifolia*, A1 - A4 – *R. alba*, *R. damascena* cultivars: I1 - I2 – 'Iskra', Y1 - Y2 – 'Yanina', E1 - E2 – 'Eleina', SV1 - SV2 – 'Svezhen'; L – Ladder 50 – 1500 bp

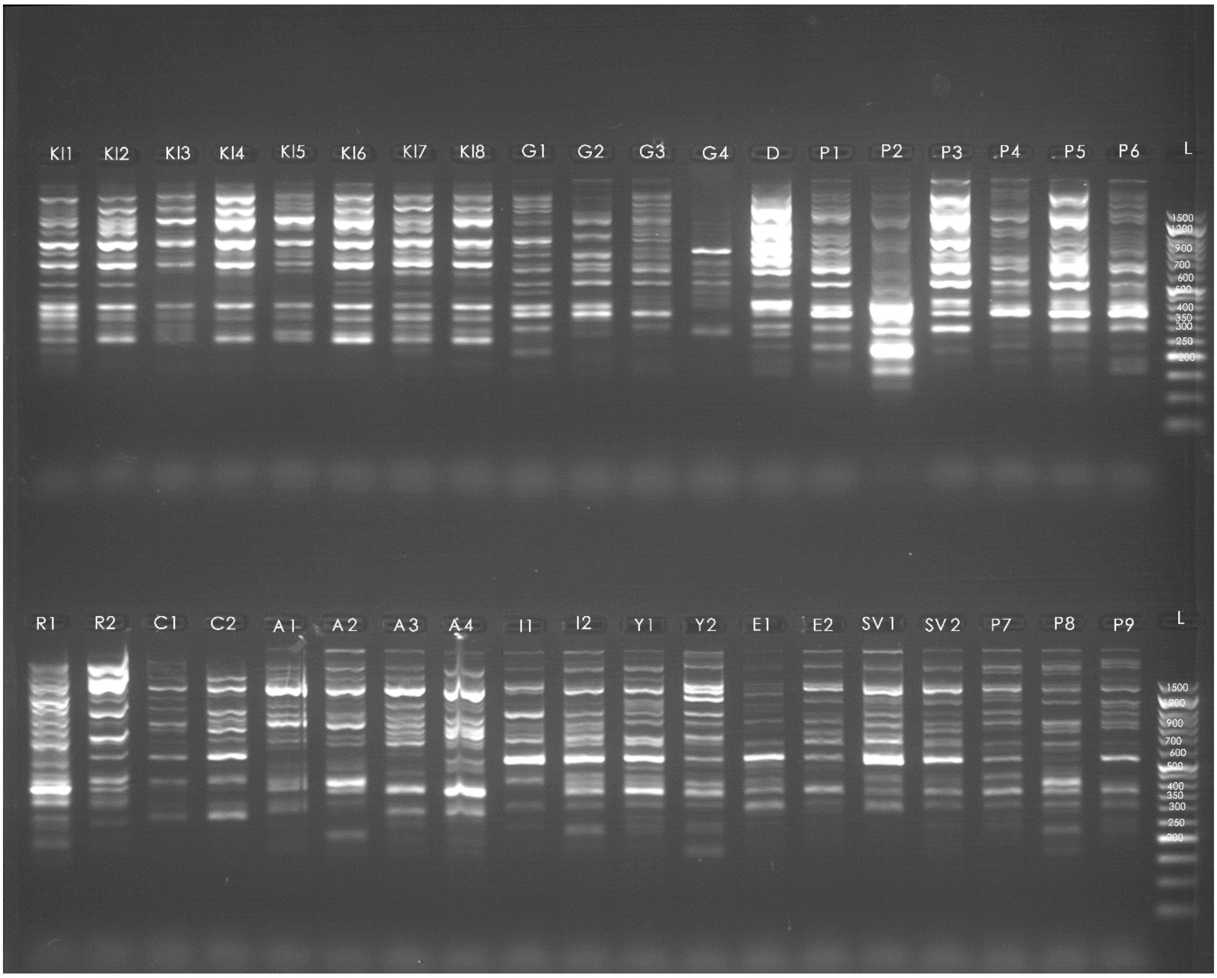

Subfigure 5. Molecular profiles of 38 *Rosa* accessions in SCoT 12 primer. Lanes marked as 1 to 38 which represent the accessions according to serial numbers in Kll - Kl8 – *Rosa* sp., G1 - G4, D – *R. gallica*, P1 - P9 – *R. damascena* 'Population 5', R1 - R2 – *Rosa* 'Raduga', C1 - C2 – *R. centifolia*, A1 - A4 – *R. alba*, *R. damascena* cultivars: I1 - I2 – 'Iskra', Y1 - Y2 – 'Yanina', E1 - E2 – 'Eleina', SV1 - SV2 – 'Svezhen'; L – Ladder 50 – 1500 bp

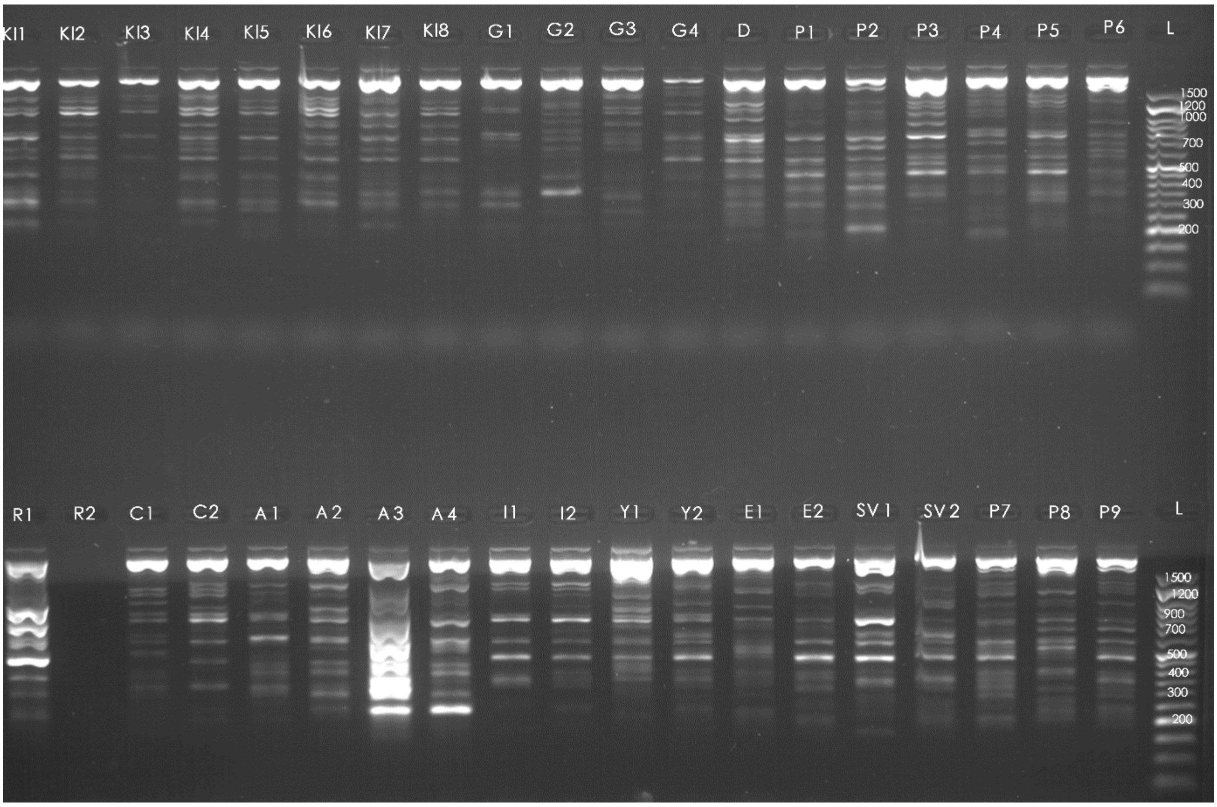

Subfigure 6. Molecular profiles of 38 *Rosa* accessions in SCoT 13 primer. Lanes marked as 1 to 38 which represent the accessions according to serial numbers in Kll - Kl8 – *Rosa* sp., G1 - G4, D – *R. gallica*, P1 - P9 – *R. damascena* 'Population 5', R1 - R2 – *Rosa* 'Raduga', C1 - C2 – *R. centifolia*, A1 - A4 – *R. alba*, *R. damascena* cultivars: I1 - I2 – 'Iskra', Y1 - Y2 – 'Yanina', E1 - E2 – 'Eleina', SV1 - SV2 – 'Svezhen'; L – Ladder 50 – 1500 bp

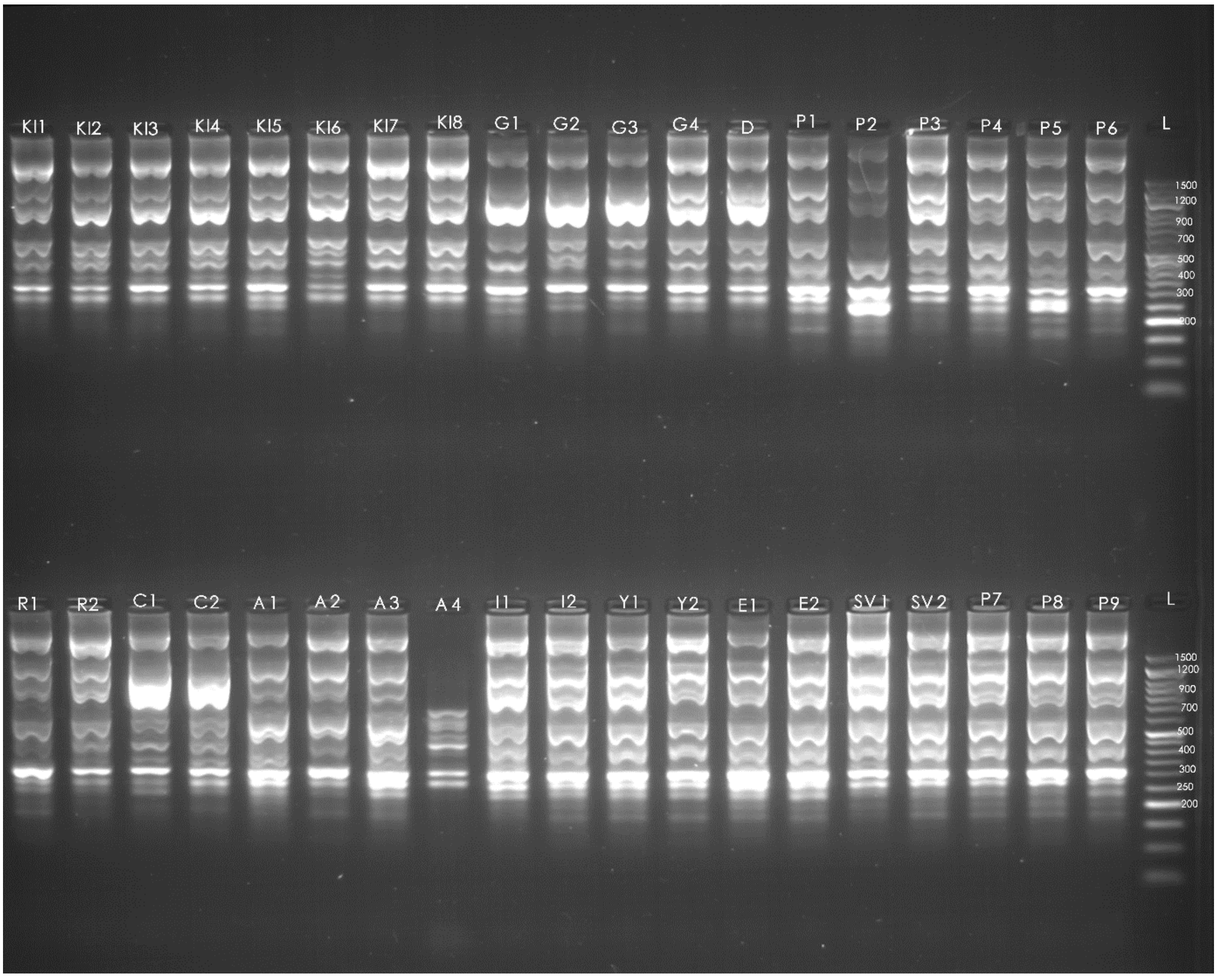

Subfigure 7. Molecular profiles of 38 *Rosa* accessions in SCoT 15 primer. Lanes marked as 1 to 38 which represent the accessions according to serial numbers in Kl1 - Kl8 – *Rosa* sp., G1 - G4, D – *R. gallica*, P1 - P9 – *R. damascena* 'Population 5', R1 - R2 – *Rosa* 'Raduga', C1 - C2 – *R. centifolia*, A1 - A4 – *R. alba*, *R. damascena* cultivars: I1 - I2 – 'Iskra', Y1 - Y2 – 'Yanina', E1 - E2 – 'Eleina', SV1 - SV2 – 'Svezhen'; L – Ladder 50 – 1500 bp

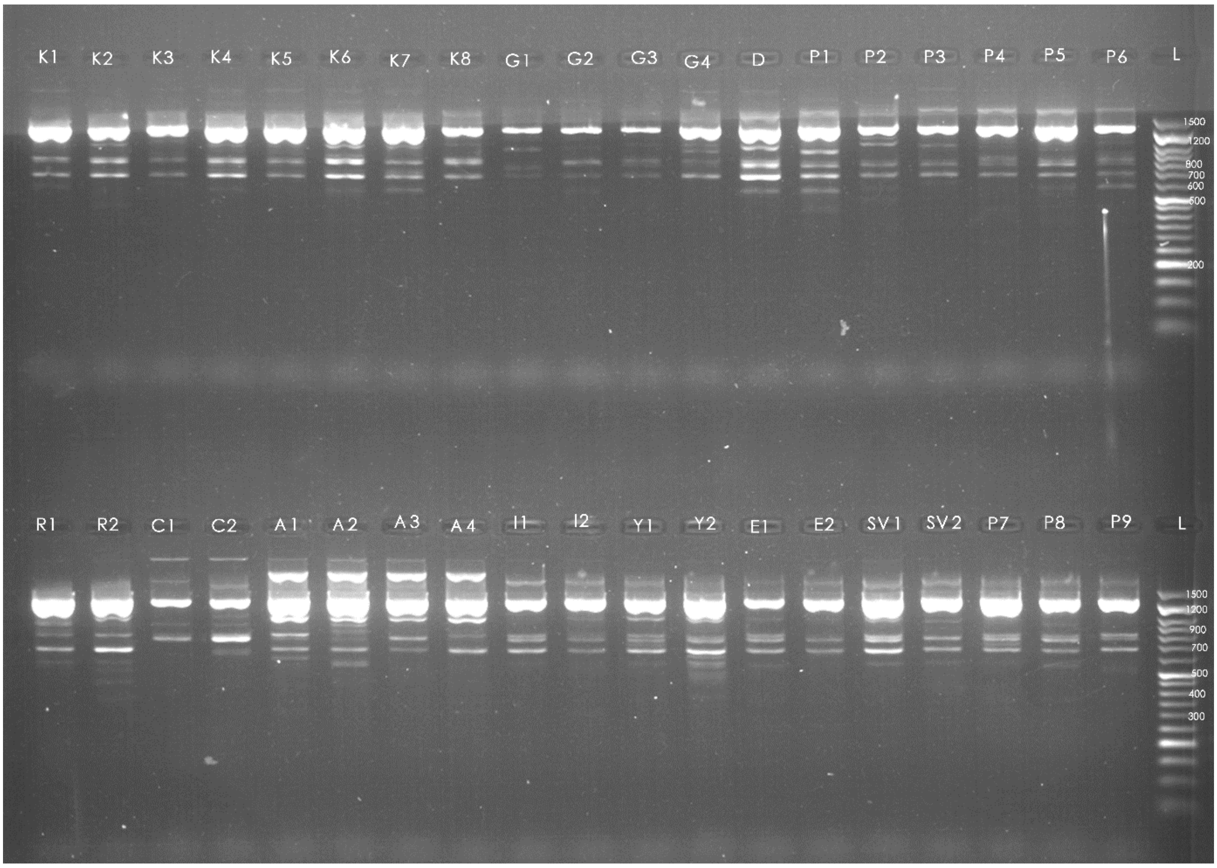

Subfigure 8. Molecular profiles of 38 *Rosa* accessions in SCoT 17 primer. Lanes marked as 1 to 38 which represent the accessions according to serial numbers in Kll - Kl8 – *Rosa* sp., G1 - G4, D – *R. gallica*, P1 - P9 – *R. damascena* 'Population 5', R1 - R2 – *Rosa* 'Raduga', C1 - C2 – *R. centifolia*, A1 - A4 – *R. alba*, *R. damascena* cultivars: I1 - I2 – 'Iskra', Y1 - Y2 – 'Yanina', E1 - E2 – 'Eleina', SV1 - SV2 – 'Svezhen'; L – Ladder 50 – 1500 bp

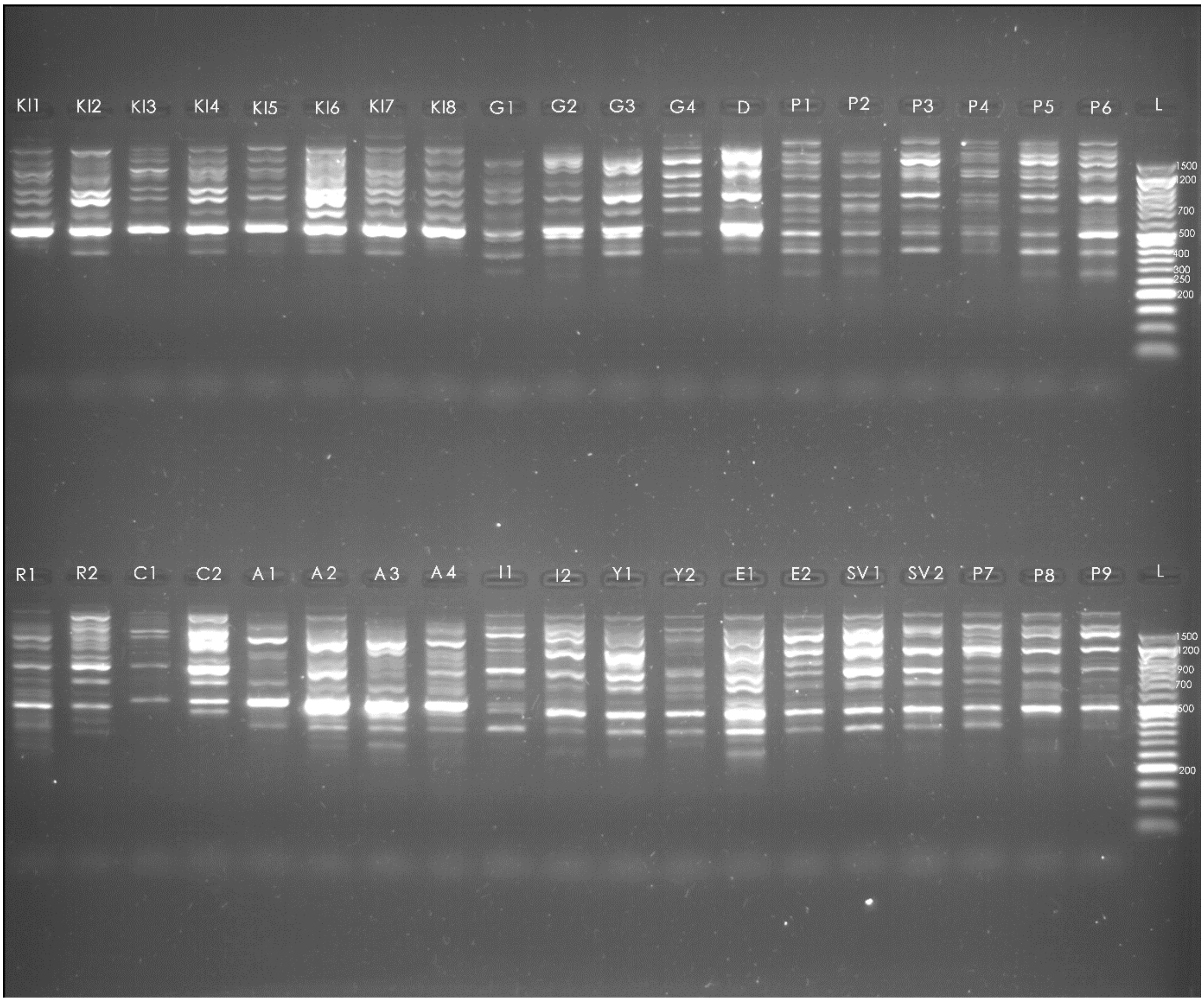

Subfigure 9. Molecular profiles of 38 *Rosa* accessions in SCoT 19 primer. Lanes marked as 1 to 38 which represent the accessions according to serial numbers in Kll - Kl8 – *Rosa* sp., G1 - G4, D – *R. gallica*, P1 - P9 – *R. damascena* 'Population 5', R1 - R2 – *Rosa* 'Raduga', C1 - C2 – *R. centifolia*, A1 - A4 – *R. alba*, *R. damascena* cultivars: I1 - I2 – 'Iskra', Y1 - Y2 – 'Yanina', E1 - E2 – 'Eleina', SV1 - SV2 – 'Svezhen'; L – Ladder 50 – 1500 bp

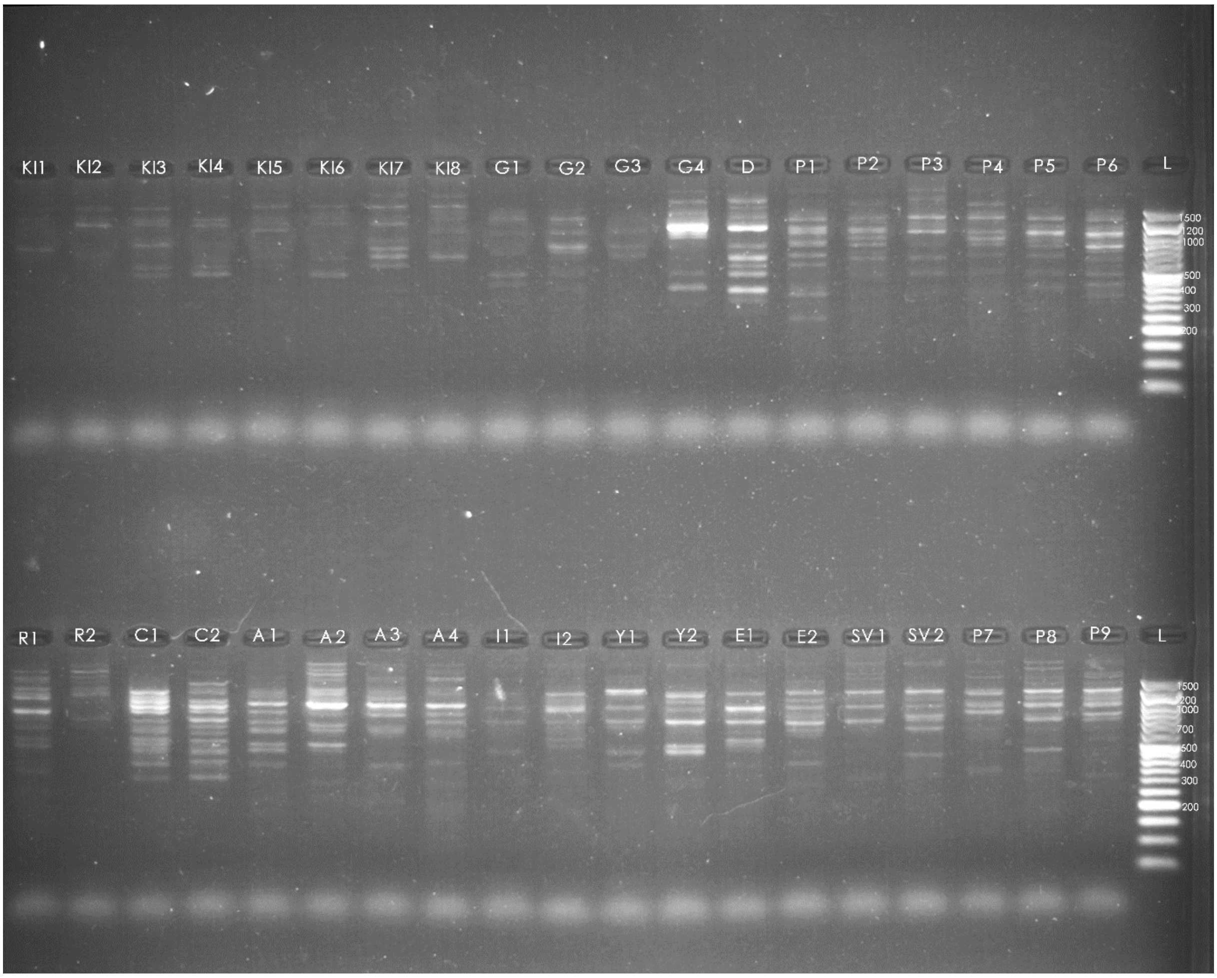

Subfigure 10. Molecular profiles of 38 *Rosa* accessions in SCoT 21 primer. Lanes marked as 1 to 38 which represent the accessions according to serial numbers in Kll - Kl8 – *Rosa* sp., G1 - G4, D – *R. gallica*, P1 - P9 – *R. damascena* 'Population 5', R1 - R2 – *Rosa* 'Raduga', C1 - C2 – *R. centifolia*, A1 - A4 – *R. alba*, *R. damascena* cultivars: I1 - I2 – 'Iskra', Y1 - Y2 – 'Yanina', E1 - E2 – 'Eleina', SV1 - SV2 – 'Svezhen'; L – Ladder 50 – 1500 bp

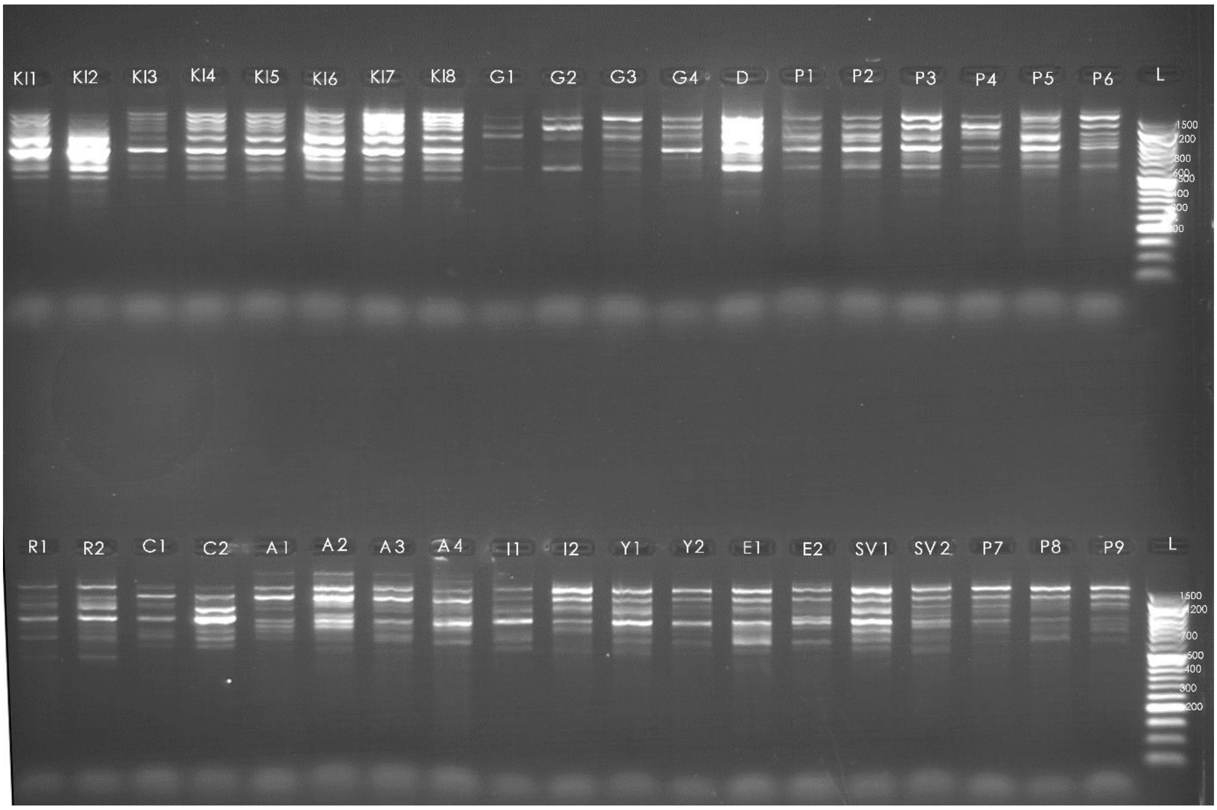

Subfigure 11. Molecular profiles of 38 *Rosa* accessions in SCoT 22 primer. Lanes marked as 1 to 38 which represent the accessions according to serial numbers in Kl1 - Kl8 – *Rosa* sp., G1 - G4, D – *R. gallica*, P1 - P9 – *R. damascena* 'Population 5', R1 - R2 – *Rosa* 'Raduga', C1 - C2 – *R. centifolia*, A1 - A4 – *R. alba*, *R. damascena* cultivars: I1 - I2 – 'Iskra', Y1 - Y2 – 'Yanina', E1 - E2 – 'Eleina', SV1 - SV2 – 'Svezhen'; L – Ladder 50 – 1500 bp

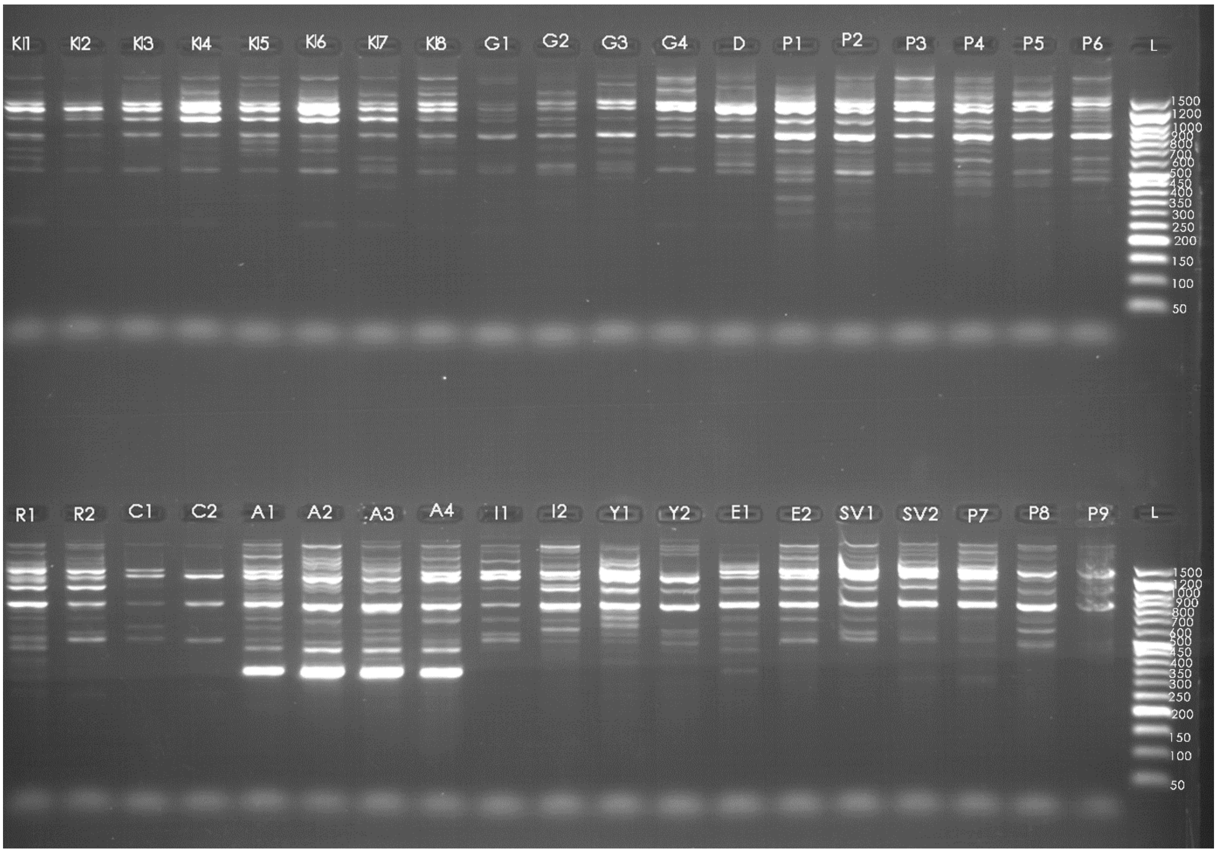

Subfigure 12. Molecular profiles of 38 *Rosa* accessions in SCoT 25 primer. Lanes marked as 1 to 38 which represent the accessions according to serial numbers in Kl1 - Kl8 – *Rosa* sp., G1 - G4, D – *R. gallica*, P1 - P9 – *R. damascena* 'Population 5', R1 - R2 – *Rosa* 'Raduga', C1 - C2 – *R. centifolia*, A1 - A4 – *R. alba*, *R. damascena* cultivars: I1 - I2 – 'Iskra', Y1 - Y2 – 'Yanina', E1 - E2 – 'Eleina', SV1 - SV2 – 'Svezhen'; L – Ladder 50 – 1500 bp

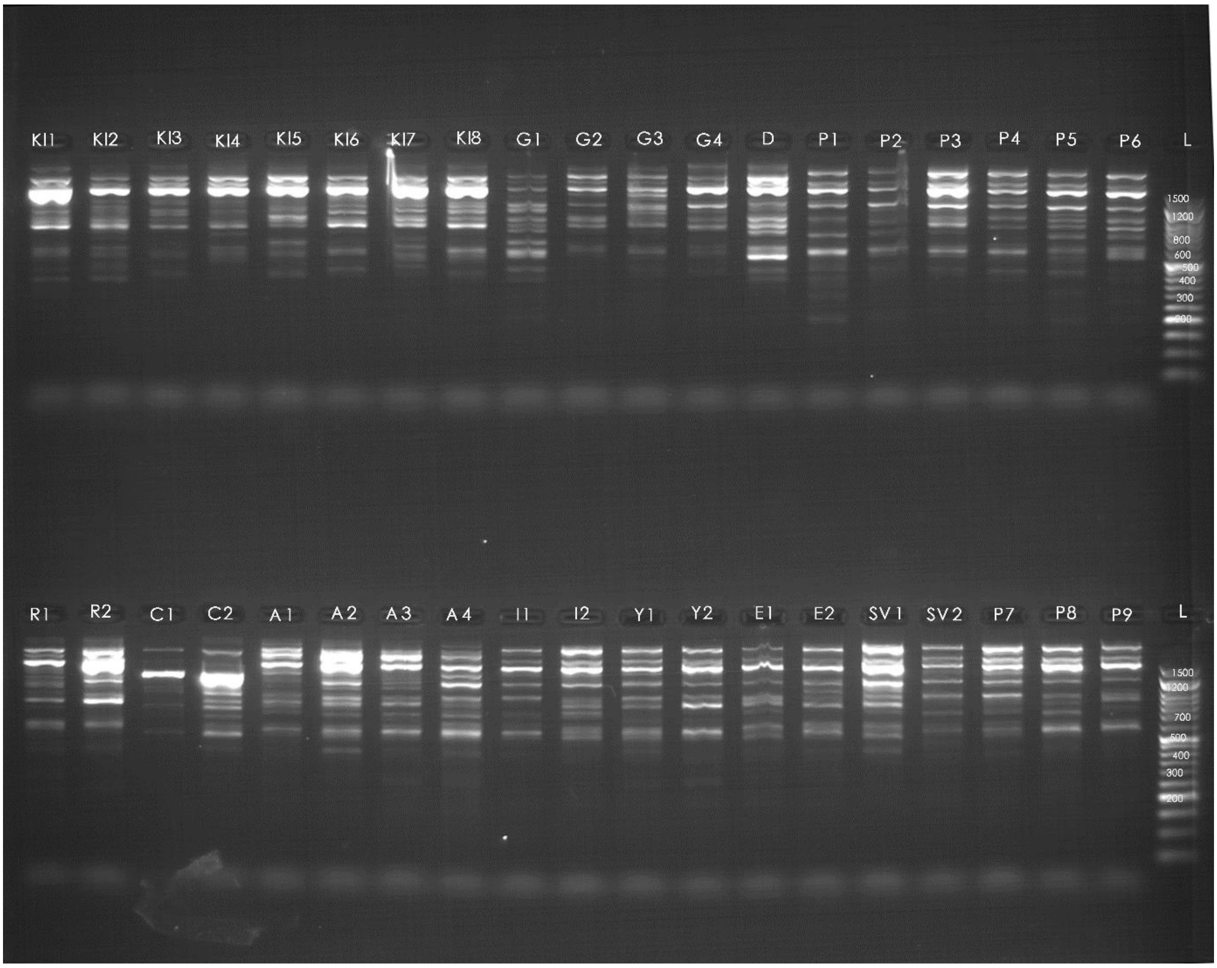

Subfigure 13. Molecular profiles of 38 *Rosa* accessions in SCoT 31 primer. Lanes marked as 1 to 38 which represent the accessions according to serial numbers in Kl1 - Kl8 – *Rosa* sp., G1 - G4, D – *R. gallica*, P1 - P9 – *R. damascena* 'Population 5', R1 - R2 – *Rosa* 'Raduga', C1 - C2 – *R. centifolia*, A1 - A4 – *R. alba*, *R. damascena* cultivars: I1 - I2 – 'Iskra', Y1 - Y2 – 'Yanina', E1 - E2 – 'Eleina', SV1 - SV2 – 'Svezhen'; L – Ladder 50 – 1500 bp

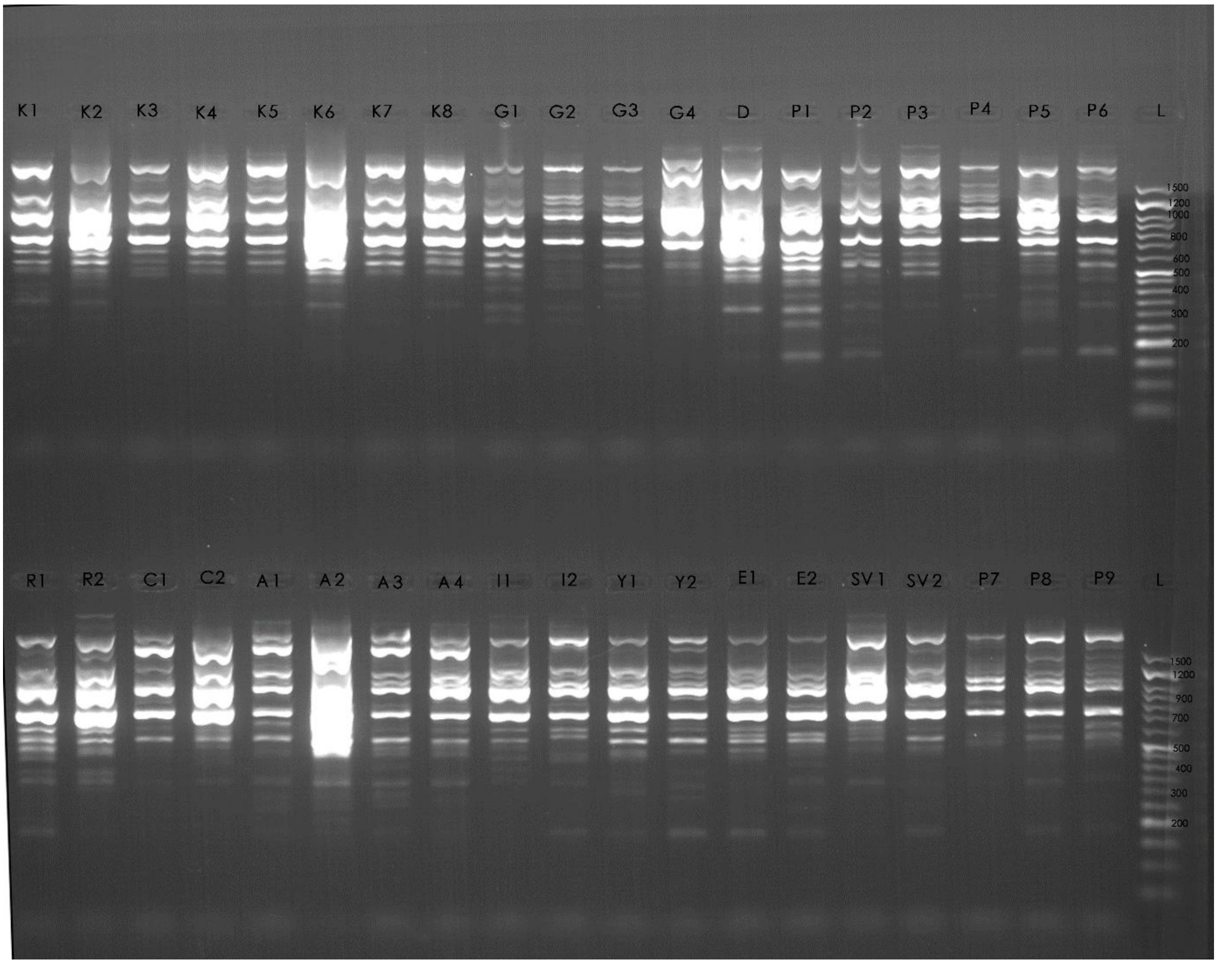

Subfigure 14. Molecular profiles of 38 *Rosa* accessions in SCoT 33 primer. Lanes marked as 1 to 38 which represent the accessions according to serial numbers in Kl1 - Kl8 – *Rosa* sp., G1 - G4, D – *R. gallica*, P1 - P9 – *R. damascena* 'Population 5', R1 - R2 – *Rosa* 'Raduga', C1 - C2 – *R. centifolia*, A1 - A4 – *R. alba*, *R. damascena* cultivars: I1 - I2 – 'Iskra', Y1 - Y2 – 'Yanina', E1 - E2 – 'Eleina', SV1 - SV2 – 'Svezhen'; L – Ladder 50 – 1500 bp

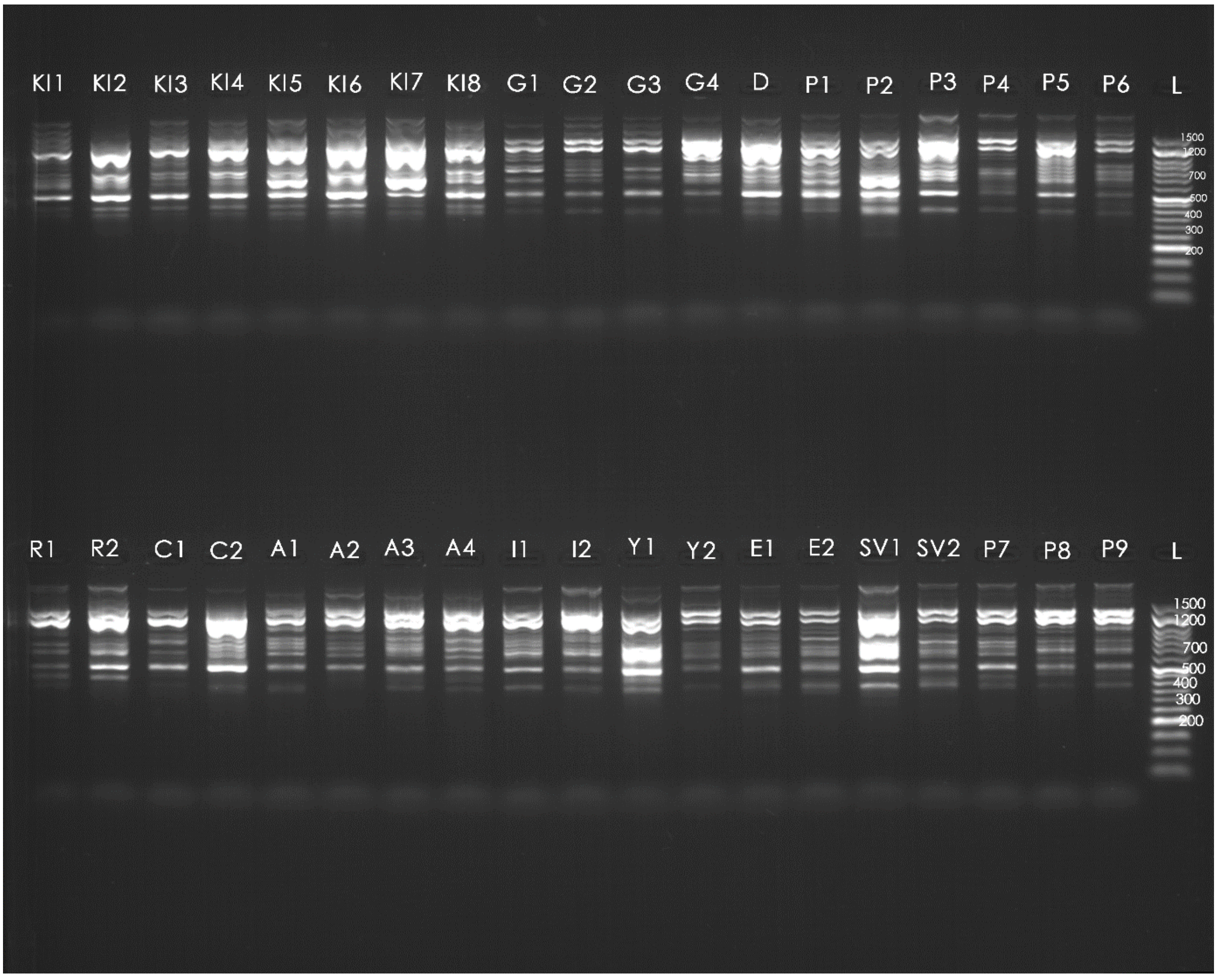

Subfigure 15. Molecular profiles of 38 *Rosa* accessions in SCoT 36 primer. Lanes marked as 1 to 38 which represent the accessions according to serial numbers in Kll - Kl8 – *Rosa* sp., G1 - G4, D – *R. gallica*, P1 - P9 – *R. damascena* 'Population 5', R1 - R2 – *Rosa* 'Raduga', C1 - C2 – *R. centifolia*, A1 - A4 – *R. alba*, *R. damascena* cultivars: I1 - I2 – 'Iskra', Y1 - Y2 – 'Yanina', E1 - E2 – 'Eleina', SV1 - SV2 – 'Svezhen'; L – Ladder 50 – 1500 bp
