# Supplementary material for: Assessment of Genetic Diversity and Population Structure in Oil-Bearing Rose Genotypes Using Start Codon-Targeted (SCoT) Markers
Source: Plants (Basel). 2026 Mar 1;15(5):761. doi: 10.3390/plants15050761 (PMC12986590; doi:10.3390/plants15050761)
Supplement: Supplementary file 1 [file plants-15-00761-s001.zip › Table S1.pdf]

Table S1. Genotype-specific loci identified in Bulgarian cultivars of *Rosa damascena* - 'Iskra', I1-I2; 'Yanina', Y1-Y2; 'Eleina' E1-E2,'Svezhen', SV1-SV2

ID	Primers	bp	ID	Primers	bp
E1	SCoT 2	1600	E1	SCoT 19	300
SV1,SV2	SCoT 3	1800	I1	SCoT 21	1900
SV1, SV2	SCoT 3	1900	E1	SCoT 25	300
SV1	SCoT 3	1700	E1, E2	SCoT 25	350
Y1	SCoT 12	150	E1, E2	SCoT 25	450
Y2	SCoT 12	250	I1	SCoT 25	1300
Y2	SCoT 17	450	Y2	SCoT 31	250
Y1	SCoT 17	750	E1, E2	SCoT 33	450
