# Supplementary material for: Assessment of Genetic Diversity and Population Structure in Oil-Bearing Rose Genotypes Using Start Codon-Targeted (SCoT) Markers
Source: Plants (Basel). 2026 Mar 1;15(5):761. doi: 10.3390/plants15050761 (PMC12986590; doi:10.3390/plants15050761)
Supplement: Supplementary file 1 [file plants-15-00761-s001.zip › Table S2.pdf]

	Kl1	Kl2	Kl3	Kl4	Kl5	Kl6	Kl7	Kl8	G1	G2	G3	G4	D	P1	P2	P3	P4	P5	P6	P7	P8	P9	R1	R2	C1	C2	A1	A2	A3	A4	I1	I2	Y1	Y2	E1	E2	SV1	SV2
Kl1	****	0.8936	0.8638	0.8723	0.8596	0.8979	0.8766	0.8894	0.7489	0.8170	0.7277	0.7234	0.7787	0.7149	0.7362	0.7489	0.7277	0.7489	0.7106	0.7064	0.7234	0.7234	0.7574	0.8000	0.7277	0.7362	0.7191	0.7447	0.7574	0.7489	0.7362	0.7149	0.7362	0.7021	0.7362	0.7574	0.7227	0.7234
Kl2	0.1125	****	0.8766	0.8936	0.8553	0.8936	0.8638	0.8511	0.7532	0.7617	0.6979	0.7277	0.7660	0.6681	0.7064	0.7362	0.7149	0.7191	0.6723	0.7021	0.6766	0.7021	0.7277	0.7787	0.7319	0.7404	0.7064	0.7064	0.7191	0.7191	0.7319	0.7021	0.7404	0.7064	0.7149	0.7447	0.7149	0.7106
Kl3	0.1464	0.1317	****	0.8723	0.8851	0.8809	0.8426	0.8383	0.7574	0.7830	0.7191	0.7489	0.7447	0.6809	0.7191	0.7404	0.7362	0.7574	0.6851	0.6979	0.6894	0.7319	0.7574	0.7404	0.7617	0.7617	0.7021	0.7106	0.7149	0.7234	0.7362	0.7234	0.7447	0.7191	0.7191	0.7234	0.7021	0.6979
Kl4	0.1366	0.1125	0.1366	****	0.9106	0.8638	0.8511	0.8638	0.7489	0.7830	0.7191	0.7489	0.7702	0.6894	0.7021	0.7489	0.7277	0.7319	0.6681	0.7149	0.7149	0.7234	0.7234	0.7574	0.7277	0.7191	0.7191	0.7106	0.7234	0.7234	0.7532	0.7149	0.7277	0.7191	0.7277	0.7404	0.7191	0.7319
Kl5	0.1513	0.1563	0.1220	0.0936	****	0.8596	0.8298	0.8766	0.7702	0.7872	0.7489	0.7532	0.7660	0.6936	0.7149	0.7532	0.7149	0.7191	0.6653	0.6936	0.7191	0.7277	0.7532	0.7872	0.7234	0.7234	0.6979	0.7234	0.7362	0.7447	0.7489	0.7362	0.7404	0.7149	0.7234	0.7191	0.7234	0.7191
Kl6	0.1077	0.1125	0.1269	0.1464	0.1513	****	0.8681	0.8723	0.7660	0.7745	0.7277	0.7404	0.7702	0.6894	0.7106	0.7404	0.7277	0.7489	0.6936	0.6809	0.6809	0.7064	0.7830	0.7830	0.7106	0.7277	0.7447	0.7617	0.7660	0.7574	0.7447	0.7234	0.7532	0.7191	0.7277	0.7489	0.7106	0.6979
Kl7	0.1317	0.1464	0.1713	0.1613	0.1866	0.1415	****	0.8851	0.7617	0.7702	0.7149	0.7191	0.8085	0.7106	0.7234	0.7532	0.7489	0.7787	0.7234	0.7277	0.7191	0.7106	0.7617	0.8043	0.7404	0.7319	0.7234	0.7574	0.7617	0.7362	0.7234	0.7277	0.7574	0.7319	0.7404	0.7532	0.7404	0.7447
Kl8	0.1117	0.1613	0.1764	0.1464	0.1317	0.1366	0.1220	****	0.7489	0.7830	0.7447	0.7319	0.7957	0.7064	0.7106	0.7574	0.7532	0.7404	0.7021	0.6979	0.7234	0.7149	0.7660	0.8000	0.7191	0.7191	0.7021	0.7277	0.7489	0.7404	0.7362	0.7149	0.7532	0.7106	0.7277	0.7319	0.7277	0.7149
G1	0.2891	0.2834	0.2778	0.2891	0.2611	0.2666	0.2722	0.2891	****	0.8128	0.7660	0.7191	0.7915	0.7191	0.7319	0.7277	0.7064	0.7191	0.6979	0.6766	0.7021	0.7106	0.7021	0.7191	0.7064	0.7404	0.6979	0.7064	0.7191	0.7191	0.7489	0.7362	0.7660	0.7149	0.7319	0.7362	0.7149	0.7191
G2	0.2021	0.2722	0.2446	0.2446	0.2392	0.2556	0.2611	0.2446	0.2073	****	0.8170	0.7787	0.7489	0.7106	0.6979	0.7532	0.7745	0.7021	0.6979	0.7106	0.7227	0.7362	0.7021	0.7362	0.7064	0.7149	0.6894	0.6894	0.7191	0.7021	0.7489	0.7277	0.7234	0.6979	0.7489	0.7532	0.7064	0.7191
G3	0.3179	0.3597	0.3297	0.3297	0.2891	0.3178	0.3356	0.2948	0.2666	0.2021	****	0.7489	0.7277	0.6979	0.6936	0.7319	0.7447	0.6894	0.7021	0.6979	0.6894	0.7064	0.6894	0.7064	0.6681	0.6851	0.6420	0.6596	0.6553	0.6638	0.7532	0.7489	0.7021	0.6851	0.7106	0.7234	0.7106	0.7234
G4	0.3238	0.3179	0.2891	0.2891	0.2834	0.3005	0.3297	0.3121	0.3297	0.2501	0.2891	****	0.7319	0.7021	0.7149	0.7277	0.7404	0.6936	0.7149	0.6766	0.7021	0.7191	0.7447	0.7277	0.6894	0.6979	0.7149	0.6723	0.6936	0.6681	0.7149	0.7277	0.7149	0.6894	0.6979	0.7106	0.7064	0.7021
D	0.2501	0.2666	0.2948	0.2611	0.2666	0.2611	0.2126	0.2285	0.2338	0.2891	0.3179	0.3121	****	0.7489	0.7617	0.7489	0.7277	0.7404	0.7277	0.7064	0.7404	0.7319	0.7574	0.7830	0.7021	0.7106	0.7191	0.7532	0.7319	0.7319	0.7277	0.7319	0.7532	0.7191	0.7532	0.7745	0.7191	0.7319
P1	0.3356	0.4033	0.3844	0.3720	0.3658	0.3720	0.3416	0.3476	0.3297	0.3416	0.3597	0.3536	0.2891	****	0.8085	0.7787	0.7830	0.8043	0.7830	0.7277	0.7787	0.7787	0.7447	0.7617	0.6383	0.6468	0.6723	0.6979	0.6936	0.6851	0.7234	0.7617	0.7489	0.7319	0.7745	0.7617	0.7149	0.7447
P2	0.3063	0.3476	0.3297	0.3536	0.3356	0.3416	0.3238	0.3416	0.3121	0.3597	0.3658	0.3356	0.2722	0.2126	****	0.8255	0.7702	0.7915	0.8213	0.7745	0.8255	0.8000	0.7830	0.7660	0.6511	0.6681	0.7106	0.7106	0.7149	0.6809	0.7447	0.7745	0.7617	0.7532	0.7872	0.7915	0.7617	0.7830
P3	0.2891	0.3063	0.3005	0.2891	0.2834	0.3005	0.2834	0.2778	0.3179	0.2834	0.3121	0.3179	0.2891	0.2501	0.1917	****	0.7915	0.8298	0.7489	0.7957	0.8213	0.8043	0.7532	0.7617	0.7234	0.7064	0.6809	0.6894	0.6936	0.6851	0.8000	0.8128	0.7915	0.7660	0.7660	0.7787	0.8000	0.8128
P4	0.3179	0.3356	0.3063	0.3179	0.3356	0.3179	0.2891	0.2834	0.3476	0.2556	0.2948	0.3005	0.3179	0.2446	0.2611	0.2338	****	0.8085	0.7872	0.7574	0.7830	0.7745	0.7319	0.7404	0.6596	0.6681	0.7191	0.7191	0.7234	0.6723	0.7617	0.7915	0.7617	0.7447	0.7787	0.7830	0.7447	0.7449
P5	0.2891	0.3297	0.2778	0.3121	0.3297	0.2891	0.2501	0.3005	0.3297	0.3536	0.3720	0.3658	0.3005	0.2178	0.2338	0.1866	0.2126	****	0.8511	0.7787	0.7702	0.7617	0.7362	0.7277	0.7149	0.6979	0.6638	0.6809	0.6766	0.6681	0.7660	0.7872	0.7830	0.7745	0.8000	0.7787	0.7489	0.7702
P6	0.3416	0.3970	0.3782	0.4033	0.4226	0.3658	0.3238	0.3536	0.3597	0.3597	0.3536	0.3356	0.3179	0.2446	0.1969	0.2891	0.2392	0.1613	****	0.7745	0.7915	0.7830	0.7234	0.7234	0.6511	0.6511	0.6851	0.6851	0.6638	0.6638	0.7532	0.7745	0.7447	0.7617	0.7787	0.7660	0.7362	0.7489
P7	0.3476	0.3536	0.3597	0.3356	0.3658	0.3844	0.3179	0.3597	0.3907	0.3416	0.3597	0.3907	0.3476	0.3179	0.2556	0.2285	0.2778	0.2501	0.2556	****	0.8128	0.8213	0.6851	0.7447	0.6723	0.6809	0.6383	0.6894	0.6851	0.6596	0.7660	0.7447	0.7234	0.7234	0.7660	0.7702	0.7660	0.7872
P8	0.3238	0.3907	0.3720	0.3356	0.3297	0.3844	0.3297	0.3238	0.3536	0.3179	0.3720	0.3536	0.3005	0.2501	0.1917	0.1969	0.2446	0.2611	0.2338	0.2073	****	0.8553	0.7532	0.7702	0.6894	0.6723	0.6979	0.7319	0.6936	0.7021	0.7574	0.7702	0.7574	0.7660	0.7489	0.7787	0.8000	0.8128
P9	0.3238	0.3536	0.3121	0.3238	0.3179	0.3476	0.3416	0.3356	0.3416	0.3063	0.3476	0.3297	0.3121	0.2501	0.2231	0.2178	0.2556	0.2722	0.2446	0.1969	0.1563	****	0.7532	0.7617	0.6809	0.6723	0.6638	0.6979	0.6681	0.6851	0.7830	0.7872	0.7660	0.7489	0.7574	0.7617	0.7574	0.7872
R1	0.2778	0.3179	0.2778	0.3238	0.2834	0.2446	0.2722	0.2666	0.3536	0.3536	0.3720	0.2948	0.2778	0.2948	0.2446	0.2834	0.3121	0.3063	0.3238	0.3782	0.2834	0.2834	****	0.8638	0.6979	0.7064	0.7489	0.7660	0.7277	0.7106	0.7064	0.7277	0.7234	0.7064	0.7574	0.7532	0.7404	0.7617

R2	0.2231	0.2501	0.3005	0.2778	0.2392	0.2446	0.2178	0.2231	0.3297	0.3063	0.3476	0.3179	0.2446	0.2722	0.2666	0.2722	0.3005	0.3179	0.3238	0.2948	0.2611	0.2722	0.1464	****	0.7149	0.7149	0.7404	0.7574	0.7447	0.7277	0.7660	0.7532	0.7489	0.7404	0.7660	0.7872	0.7915	0.7957
C1	0.3179	0.3121	0.2722	0.3179	0.3416	0.3416	0.3005	0.3297	0.3476	0.3476	0.4033	0.3720	0.3536	0.4490	0.4291	0.3238	0.4162	0.3356	0.4291	0.3970	0.3720	0.3844	0.3597	0.3356	****	0.9149	0.7106	0.6681	0.6723	0.7149	0.7277	0.7319	0.7447	0.7447	0.6936	0.7149	0.7191	0.7234
C2	0.3063	0.3005	0.2722	0.3297	0.3179	0.3179	0.3121	0.3297	0.3005	0.3356	0.3782	0.3597	0.3416	0.4357	0.4033	0.3476	0.4033	0.3597	0.4291	0.3844	0.3970	0.3970	0.3476	0.3356	0.0889	****	0.7277	0.7106	0.6894	0.7489	0.7447	0.7234	0.7362	0.7277	0.7021	0.7149	0.7191	0.7234
A1	0.3297	0.3476	0.3536	0.3297	0.2948	0.2948	0.3238	0.3536	0.3597	0.3720	0.4423	0.3356	0.3297	0.3970	0.3416	0.3844	0.3297	0.4097	0.3782	0.4490	0.3597	0.4097	0.2891	0.3005	0.3416	0.3179	****	0.8553	0.8596	0.8085	0.7362	0.7064	0.7106	0.7021	0.7532	0.7660	0.7277	0.7319
A2	0.2948	0.3476	0.3416	0.3416	0.2722	0.2722	0.2778	0.3179	0.3297	0.3297	0.4162	0.3970	0.2834	0.3597	0.3416	0.3720	0.3297	0.3844	0.3782	0.3720	0.3121	0.3597	0.2666	0.2778	0.4033	0.3461	0.1563	****	0.8766	0.8340	0.7277	0.7234	0.7362	0.7447	0.7872	0.7745	0.7277	0.7149
A3	0.2778	0.3297	0.3356	0.3238	0.2666	0.2666	0.2722	0.2891	0.3297	0.3536	0.4226	0.3658	0.3121	0.3658	0.3356	0.3658	0.3238	0.3907	0.4097	0.3782	0.3658	0.4033	0.3179	0.2948	0.3970	0.3720	0.1513	0.1317	****	0.8468	0.6979	0.6851	0.7064	0.6723	0.7660	0.7872	0.7149	0.7277
A4	0.2891	0.3297	0.3238	0.3238	0.2778	0.2778	0.3063	0.3005	0.2891	0.2891	0.4097	0.4033	0.3121	0.3728	0.3844	0.3782	0.3970	0.4033	0.4097	0.4162	0.3536	0.3782	0.3416	0.3179	0.3356	0.2891	0.2126	0.1815	0.1663	****	0.7234	0.7021	0.7149	0.6894	0.7064	0.7362	0.7064	0.7106
I1	0.3063	0.3121	0.3063	0.2834	0.2948	0.2948	0.3238	0.3063	0.3063	0.3179	0.2834	0.3356	0.3179	0.3238	0.2948	0.2231	0.2722	0.2666	0.2834	0.2666	0.2778	0.2446	0.3476	0.2666	0.3179	0.2948	0.3063	0.3179	0.3597	0.3238	****	0.8851	0.8213	0.7957	0.8128	0.8255	0.8468	0.8340
I2	0.3356	0.3536	0.3238	0.3356	0.3238	0.3238	0.3179	0.3356	0.2666	0.3238	0.2891	0.3179	0.3121	0.2722	0.2556	0.2073	0.2338	0.2392	0.2556	0.2948	0.2611	0.2392	0.3179	0.2834	0.3121	0.3238	0.3476	0.3238	0.3782	0.3536	0.1220	****	0.8511	0.8170	0.8255	0.8213	0.8170	0.8213
Y1	0.3063	0.3005	0.2948	0.3179	0.2834	0.2834	0.2778	0.2834	0.3356	0.3597	0.3536	0.3356	0.2834	0.2891	0.2722	0.2338	0.2722	0.2446	0.2948	0.3238	0.2778	0.2666	0.3238	0.2891	0.2948	0.3063	0.3416	0.3063	0.3476	0.3356	0.1969	0.1613	****	0.8809	0.8128	0.8085	0.8043	0.8000
Y2	0.3536	0.3476	0.3297	0.3297	0.3297	0.3297	0.3121	0.3416	0.3121	0.2891	0.3782	0.3720	0.3297	0.3121	0.2834	0.2666	0.2948	0.2556	0.2722	0.3238	0.2666	0.2891	0.3476	0.3005	0.2948	0.3179	0.3536	0.2948	0.3970	0.3720	0.2285	0.2021	0.1269	****	0.8128	0.8085	0.7787	0.7830
E1	0.3063	0.3356	0.3297	0.3179	0.3179	0.3179	0.3005	0.3179	0.3063	0.2834	0.3416	0.3597	0.2834	0.2556	0.2392	0.2666	0.2501	0.2231	0.2501	0.2666	0.2891	0.2778	0.2778	0.2666	0.3658	0.3536	0.2834	0.2392	0.2666	0.3476	0.2073	0.1917	0.2073	0.2073	****	0.9106	0.7975	0.8170
E2	0.2778	0.3238	0.3238	0.3005	0.2891	0.2891	0.2834	0.3121	0.3063	0.3476	0.3238	0.3416	0.2556	0.2722	0.2338	0.2501	0.2446	0.2501	0.2666	0.2611	0.2501	0.2722	0.2834	0.2392	0.3356	0.3556	0.2666	0.2556	0.2392	0.3063	0.1917	0.1969	0.2126	0.2126	0.0936	****	0.8340	0.8553
SV1	0.3179	0.3356	0.3536	0.3297	0.3416	0.3416	0.3005	0.3179	0.3356	0.3356	0.3416	0.3476	0.3297	0.3356	0.2722	0.2231	0.2948	0.2891	0.3063	0.2666	0.2231	0.2778	0.3005	0.2338	0.3297	0.3297	0.3179	0.3179	0.3356	0.3476	0.1663	0.2021	0.2178	0.2501	0.2285	0.1815	****	0.9191
SV2	0.3238	0.3416	0.3597	0.3121	0.3597	0.3597	0.2948	0.3356	0.3297	0.3297	0.3238	0.3536	0.3121	0.2948	0.2446	0.2073	0.2891	0.2611	0.2891	0.2392	0.2073	0.2392	0.2722	0.2285	0.3238	0.3238	0.3121	0.3356	0.3173	0.3416	0.1815	0.1969	0.2231	0.2446	0.2021	0.1563	0.0843	****
