# Supplementary material for: Assessment of Genetic Diversity and Population Structure in Oil-Bearing Rose Genotypes Using Start Codon-Targeted (SCoT) Markers
Source: Plants (Basel). 2026 Mar 1;15(5):761. doi: 10.3390/plants15050761 (PMC12986590; doi:10.3390/plants15050761)
Supplement: Supplementary file 1 [file plants-15-00761-s001.zip › Table S3.Q value compositions of 38 Rosa accessions.pdf]

Table S3. Q value compositions of 38 *Rosa* accessions Kll - Kl8 – *Rosa* sp., G1 - G4, D – *R. gallica*, P1 - P9 – *R. damascena* 'Population 5', R1 - R2 – *Rosa* 'Raduga', C1 - C2 – *R. centifolia*, A1 - A4 – *R. alba*, *R. damascena* cultivars: I1 - I2 – 'Iskra', Y1 - Y2 – 'Yanina', E1 - E2 – 'Eleina', SV1 - SV2 – 'Svezhen'.

**Pop ID**	**Q1**	**Q2**	**Q3**	**Q4**	**Q5**	**Pop ID**	**Q1**	**Q2**	**Q3**	**Q4**	**Q5**
**Kl1**	0.002	0.003	0.002	0.001	0.992	**P7**	0.001	0.001	0.983	0.03	0.002
**Kl2**	0.002	0.001	0.001	0.003	0.993	**P8**	0.001	0.002	0.991	0.004	0.001
**Kl3**	0.019	0.001	0.001	0.003	0.974	**P9**	0.001	0.001	0.992	0.004	0.001
**Kl4**	0.002	0.001	0.001	0.002	0.994	**R1**	0.005	0.121	0.437	0.325	0.112
**Kl5**	0.002	0.001	0.001	0.002	0.994	**R2**	0.007	0.020	0.442	0.336	0.195
**Kl6**	0.001	0.002	0.001	0.001	0.994	**C1**	0.977	0.007	0.001	0.009	0.005
**Kl7**	0.005	0.006	0.002	0.017	0.970	**C2**	0.977	0.007	0.001	0.009	0.005
**Kl8**	0.002	0.002	0.001	0.003	0.993	**A1**	0.024	0.957	0.009	0.009	0.001
**G1**	0.006	0.002	0.003	0.968	0.021	**A2**	0.001	0.985	0.011	0.002	0.001
**G2**	0.003	0.001	0.004	0.959	0.033	**A3**	0.001	0.994	0.002	0.001	0.002
**G3**	0.002	0.001	0.009	0.984	0.005	**A4**	0.125	0.852	0.001	0.013	0.008
**G4**	0.007	0.005	0.051	0.902	0.035	**I1**	0.014	0.004	0.941	0.036	0.006
**D**	0.007	0.009	0.022	0.899	0.063	**I2**	0.014	0.004	0.941	0.036	0.006
**P1**	0.001	0.008	0.943	0.046	0.002	**Y1**	0.009	0.002	0.973	0.005	0.010
**P2**	0.001	0.004	0.990	0.002	0.002	**Y2**	0.019	0.003	0.970	0.005	0.040
**P3**	0.003	0.002	0.984	0.005	0.006	**E1**	0.002	0.026	0.966	0.005	0.001
**P4**	0.001	0.007	0.879	0.109	0.004	**E2**	0.002	0.030	0.961	0.004	0.003
**P5**	0.005	0.001	0.976	0.004	0.014	**SV1**	0.003	0.003	0.990	0.002	0.002
**P6**	0.001	0.001	0.992	0.003	0.002	**SV2**	0.003	0.003	0.990	0.002	0.002
