# Supplementary figures and images for: Assessment of Genetic Diversity and Population Structure in Oil-Bearing Rose Genotypes Using Start Codon-Targeted (SCoT) Markers
Source: Plants (Basel). 2026 Mar 1;15(5):761. doi: 10.3390/plants15050761 (PMC12986590; doi:10.3390/plants15050761)

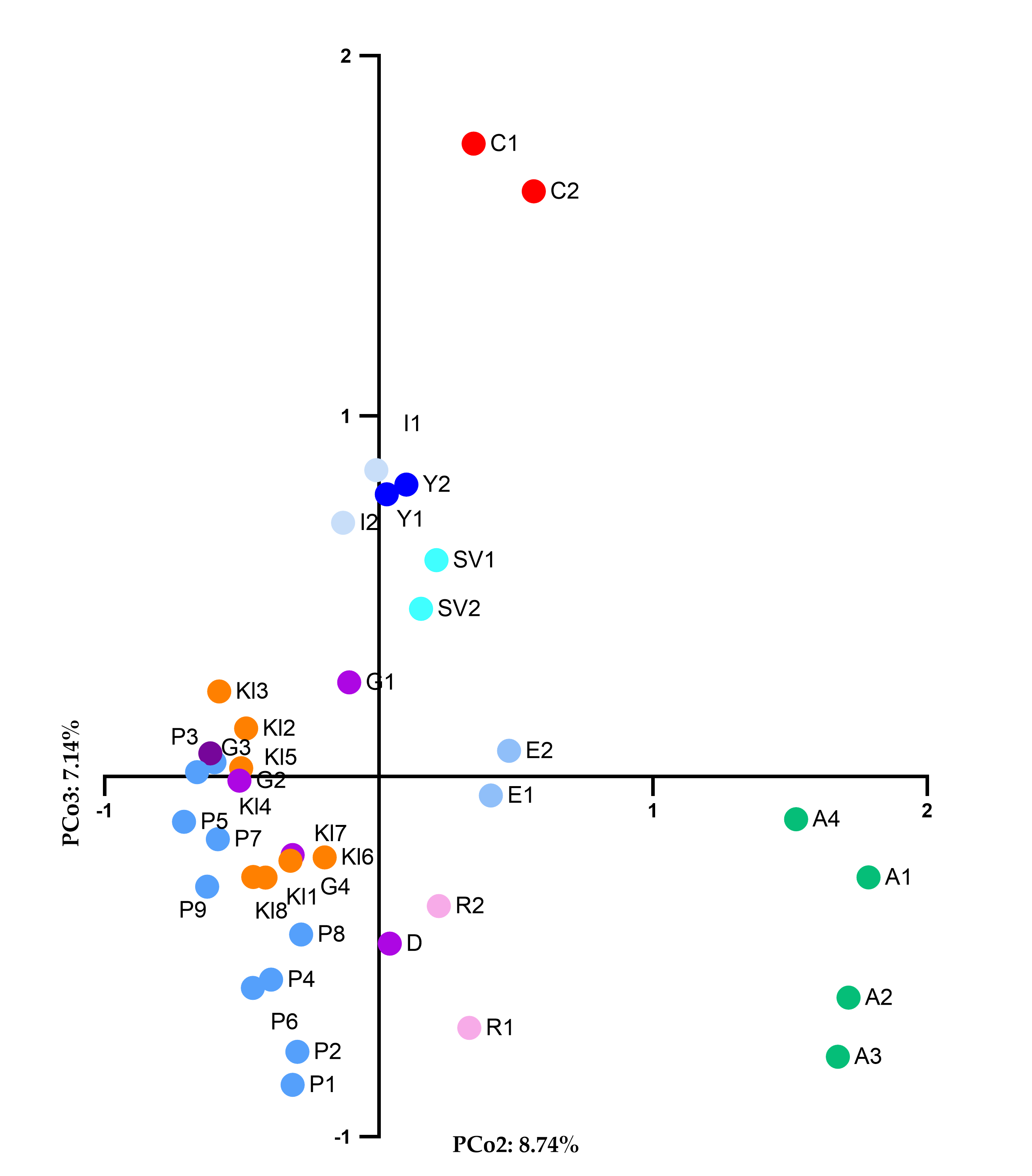

Supplement: Supplementary file 1 [file plants-15-00761-s001.zip › Figure S2. Principal coordinate analysis (PCoA) biplot of the studied 38 Rosa accessions.png]
